# Frequency-Resolved Dynamic Functional Connectivity Reveals Scale-Stable Features of Connectivity-States

**DOI:** 10.3389/fnhum.2018.00253

**Published:** 2018-06-26

**Authors:** Markus Goldhacker, Ana M. Tomé, Mark W. Greenlee, Elmar W. Lang

**Affiliations:** ^1^CIML Lab, Department of Biophysics, University of Regensburg, Regensburg, Germany; ^2^Department of Experimental Psychology, University of Regensburg, Regensburg, Germany; ^3^Departamento de Eletrónica, Telecomunicações e Informática (DETI), Instituto de Engenharia Electrónica e Telemática de Aveiro (IEETA), Universidade de Aveiro, Aveiro, Portugal

**Keywords:** dynamic functional connectivity, multivariate, empirical mode decomposition, filter-bank, multiscale, fMRI, resting-state, scale-invariance

## Abstract

Investigating temporal variability of functional connectivity is an emerging field in connectomics. Entering dynamic functional connectivity by applying sliding window techniques on resting-state fMRI (rs-fMRI) time courses emerged from this topic. We introduce frequency-resolved dynamic functional connectivity (frdFC) by means of multivariate empirical mode decomposition (MEMD) followed up by filter-bank investigations. In general, we find that MEMD is capable of generating time courses to perform frdFC and we discover that the structure of connectivity-states is robust over frequency scales and even becomes more evident with decreasing frequency. This scale-stability varies with the number of extracted clusters when applying *k*-means. We find a scale-stability drop-off from *k* = 4 to *k* = 5 extracted connectivity-states, which is corroborated by null-models, simulations, theoretical considerations, filter-banks, and scale-adjusted windows. Our filter-bank studies show that filter design is more delicate in the rs-fMRI than in the simulated case. Besides offering a baseline for further frdFC research, we suggest and demonstrate the use of scale-stability as a possible quality criterion for connectivity-state and model selection. We present first evidence showing that connectivity-states are both a multivariate, and a multiscale phenomenon. A data repository of our frequency-resolved time-series is provided.

## 1. Introduction

Functional connectivity is a key aspect in the analysis of rs-fMRI. It is based on calculating association measures—mostly Pearson correlation—between distinct regions in the brain. First attempts focused on the static case, for which whole time courses of resting-state-related brain regions were used for evaluating correlation coefficients representing the strength of their functional connections (Eguíluz et al., 2005; Lang et al., 2012). This approach resulted in many insights ranging from a small-world organization of brain graphs that are constructed from this so-called *connectome* (Bullmore and Sporns, 2009) over deviations in functional connectivity between pathological and healthy brains (Stam et al., 2007; Ma et al., 2014) to developmental changes of functional connectivity (Geerligs et al., 2014). It was also possible to identify similarities between physical systems like the *Ising-model* of a ferromagnet and functional connectivity brain networks (Fraiman et al., 2009). However, using whole time courses integrates out all temporal dependences within the connectome, resulting in static average connectivities. Recently a paradigm shift occurred toward a so-called dynamic functional connectivity (dFC), which takes into account the temporal variability of functional connections in the brain. Investigating temporal fluctuations of functional connectivity thus has received considerable attention in the last few years (Chang and Glover, 2010; Deco et al., 2011; Cribben et al., 2012; Allen et al., 2014; Calhoun et al., 2014; Kopell et al., 2014).

Allen et al. (2014) introduced a sliding-window technique applied to the time courses of independent components (ICs) resulting from a group independent component analysis (gICA) (Calhoun et al., 2001) on a very large set of subjects undergoing rs-fMRI. This technique is also employed in the present study. The idea is to track the variability of correlation matrices formed from segments of the time courses of all ICs. Shifting the window one time step further, results in a new correlation matrix for the next time step with slight changes in correlation coefficients and so on. The vast set of correlation matrices resulting from such a sliding-window approach can be condensed into several representative correlation patterns by applying *k*-means clustering Allen et al. (2014). These stable patterns, which robustly showed up as cluster-centroids, can be considered *connectivity-states* given the fact that these centroids represent very robust and almost discrete correlation patterns reflecting characteristic connectivities that the brain goes through over time, while simultaneously remaining similar between subjects.

The number of extracted clusters has to be predefined using the *k*-means algorithm. Usually it is deduced from an elbow-criterion which indicates a sudden change in a cluster similarity index that, e.g., compares the variance within the extracted clusters to the variance between them. Selecting the data inherent number of clusters in the mentioned way is usually far from objective, since a pronounced elbow is lacking most of the time. In our study, we introduce a novel way of resolving this issue by exploiting the novel finding of scale invariance of connectivity-states. Scale invariance or scale-stability has been shown to be an inherent feature of rs-fMRI data (Eguíluz et al., 2005; Kitzbichler et al., 2009; Moretti and Muñoz, 2013). Scale-stability of connectivity-states should be taken into account when conducting a dFC analysis and we suggest to optimize it in terms of connectivity-state extraction. Consequently, in this study we explore the persistence of connectivity-states across frequency scales and dive into scale invariance investigations of connectivity-states.

One recent study Yaesoubi et al. (2015) investigated frdFC by applying a wavelet decomposition to the time courses resulting from gICA. We address this point by applying multivariate empirical mode decomposition (MEMD), which is a data-driven method for extracting so-called IMFs (Huang et al., 1998), which reveal inherent characteristic time scales of temporal variations of the quantities under study. Thus MEMD yields time courses corresponding to clearly separated and narrow-band frequency scales, which can be investigated individually by means of dFC. Since the frequency scales of interest are not known beforehand, starting with data-driven exploratory methods like MEMD is a natural way. Our *post hoc* analyses with filter-banks confirm that this way of approaching this kind of data is promising.

By entering frdFC by means of MEMD, we found that connectivity-states show remarkable scale invariance features, and that only the data-inherent number of connectivity-states show scale invariant features over all extracted frequency scales. Extracting more connectivity-states than present in the data results in an abrupt drop in scale invariance. We found this result both in rs-fMRI data, and in simulated connectivity data. Our findings deduced in the present manuscript yield a novel method of selecting the data inherent number of connectivity-states, or rather number of clusters, by exploiting scale invariance of rs-fMRI data. Content from one of the author's thesis (Goldhacker, 2017)^1^ has been included in this manuscript.

The structure of the manuscript is as follows. In section 2, the methodological background and pipeline are introduced. Section 3 then starts with the quantification of scale invariance of connectivity-states yielding the result for identifying the data-inherent number of connectivity-states in rsfMRI (sections 3.1 and 3.2). This approach of detecting the data-inherent number of connectivity-states and the mentioned result are corroborated with several validation steps (sections 3.3–3.6). Section 4 discusses our approach, its implications, and limitations.

## 2. Materials and methods

In this section, we deduce the applied processing pipeline. We used rs-fMRI data from the Human Connectome Project, on which gICA has been applied to. This yielded time courses for a predefined number of brain areas on a single subject level. On those time courses, MEMD has been applied resulting in IMFs on distinct and aligned frequency scales. The dFC approach has then been applied to those IMFs on distinct frequency scales yielding the proposed frdFC approach.

### 2.1. Data-set

We based our analysis on volumetric data from the preselected bundle of 100 unrelated human subjects from the S500 release of the *Human Connectome Project* (Van Essen et al., 2012), in which each subject went through four rs-fMRI sessions lasting 14min 33s resulting in 1200 volumes per session and *n* = 400 sessions in total. Data was acquired at customized 3T MRI scanners at Washington University using multi-band (factor 8) acquisition techniques (Moeller et al., 2010; Feinberg et al., 2010; Setsompop et al., 2012; Xu et al., 2012). From the different versions of the data we chose the most preprocessed data set with motion-correction, structural preprocessing, and ICA-FIX denoising (Jenkinson et al., 2002, 2012; Fischl, 2012; Glasser et al., 2013; Smith et al., 2013; Griffanti et al., 2014; Salimi-Khorshidi et al., 2014). The rs-fMRI data has a TR of 720ms and TE of 33.1ms acquired by a Gradient-echo EPI sequence. Flip angle was 52° and the FOV 208 × 180 mm with a slice thickness of 2 mm, 72 slices, and isotropic 2 mm voxel size. As additional preprocessing we applied a Gaussian smoothing kernel with a FWHM of 5 mm using SPM8 software package (http://www.fil.ion.ucl.ac.uk/spm/) and we discarded the first five scans of each session from our analysis.

### 2.2. Group-ICA

**Figure 1:**
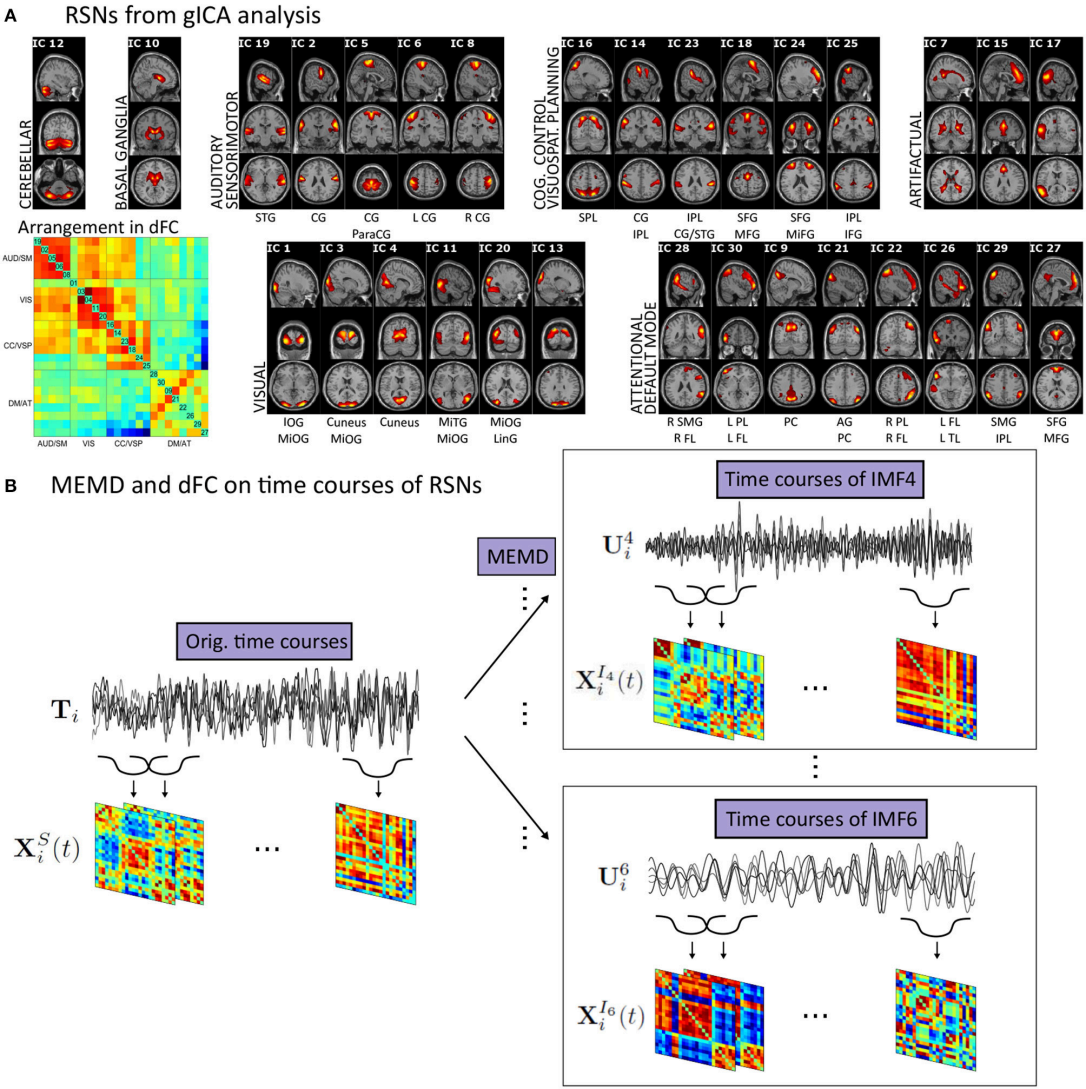
**(A)** This panel shows the results from the gICA analysis of all 400 sessions arranged in modules concerning similar correlation behavior. Activity maps represent the average over all sessions with values being *z*-transformed. The cut-off is chosen as *z*>2 and the highest value of the color-range is individually determined for each map. The arrangement of the RSNs in the dFC matrices is shown on bottom left of this panel. This correlation matrix shows the average of the dFC matrices over all time points and sessions. Note the clear segregation into functional modules. Below each RSN characteristic areas are mentioned. STG, Superior Temporal Gyrus; CG, Central Gyrus; SPL, Superior Parietal Lobule; IPL, Inferior Parietal Lobule; SFG, Superior Frontal Gyrus; MFG, Medial Frontal Gyrus; MiFG, Middle Frontal Gyrus; IFG, Inferior Frontal Gyrus; IOG, Inferior Occipital Gyrus; MiOG, Middle Occipital Gyrus; MiTG, Middle Temporal Gyrus; LinG, Lingual Gyrus; SMG, Supramarginal Gyrus; FL, Frontal Lonule; PL, Parietal Lobule; PC, Precuneus; AG, Angular Gyrus; TL, Temporal Lobule; SFG, Superior Frontal Gyrus. **(B)** The base of our approach is to decompose the time courses of all RSNs by EMD resulting in ten separate frequency scales. To both on the original time courses and the time courses of each frequency scale dFC is applied yielding sets of correlation matrices.

To apply gICA (Calhoun et al., 2001) on our data we used the GIFT toolbox (http://mialab.mrn.org/software/gift/). On the single-subject level, our data matrices **D**_*i, M*×*K*_ have time as the row dimension (*M* = 1195) and voxel-space as the column dimension (*K* = 193965). As additional preprocessing, before entering the analysis pipeline, we used variance normalization by linearly detrending and *z*-scoring each voxel time-series. At the single-subject reduction step, we extracted *m* = 45 principal components (PCs) from the data of each session. By solving the eigenvector decomposition ${\text{C}}_{i}={\text{V}}_{i}{\text{L}}_{i}{\text{V}}_{i}^{T}$ of the data-covariance matrix ${\text{C}}_{i}={\text{D}}_{i}^{T}{\text{D}}_{i}$ with **V**_*i*_ representing the matrix of PCs and **L**_*i*_ the eigenvalue matrix, a PCA allowed to substantially reduce the dimensionality of the problem. By using the *m* = 45 projections onto the PCs with the highest eigenvalues from **L**_*i*_, a *m*×*K* reduced data matrix ${\text{R}}_{i}^{\left(s\right)}$ resulted on the single-subject level. This was followed by a group reduction step, stacking all ${\text{R}}_{i}^{\left(s\right)}$ in the row domain resulting, on the group level, in an *nm*×*K* matrix **R**^**(g)**^ entering once again a PCA, in analogy to the single-subject step, and, finally, extracting 30 PCs across all subjects. All PCAs were, because of limited on-board memory, implemented with the help of an expectation maximization algorithm (Roweis, 1998). On the resulting group data set, we evaluated gICA **R**^(*g*)^ =**TS** by applying the Infomax algorithm (Bell and Sejnowski, 1995) to extract spatially independent components (ICs) **S** and by applying the ICASSO approach (Himberg et al., 2004) for component stability while repeating the ICA algorithm 10 times. Finally, ICs were extracted from our data similar to Smith et al. (2013), which ensures computability when it comes to applying the MEMD algorithm (Huang et al., 1998; Mandic et al., 2013). To come back from the group level to the single-subject level, the back-reconstruction algorithm GICA3 (Erhardt et al., 2011) was employed yielding subject specific time courses **T**_*i*_ and spatial maps **S**_*i*_. Afterwards, the spatial maps were *z*-scored. The results of the gICA analysis are illustrated in Figure 1A.

We tested our results extensively concerning stability and performed our analysis with different numbers of components. Looking at the dynamic range and low to high frequency ratio (Allen et al., 2011), which reflects spectral characteristics of the ICs, we find that the obvious artifact ICs 7, 15, and 17 have lower values in both measures (Figure S1). Additionally ICs 10, 12, and 13 have lower values in those measures than the best artifact component (IC 7). Consequently, we tested our approach by dividing the set of ICs in a more conservative, an intermediate, and a liberal set of resting-state networks (RSNs), by which we identify ICs representing functionally important networks. For the conservative set we removed ICs 10, 12, and 13, in addition to the three artifactual ICs related to vascular (IC 15), cerebro-spinal fluid (IC 7), and white matter components (IC 17) resulting in 24 ICs as RSNs. The more liberally selected set of ICs consisted of a total of 27 RSNs leaving out the artifact ICs, while the intermediate selection led us to remove IC 13 in addition to the artifactual ICs, since dFC analysis showed that this IC is very noisy resulting in a data set with 26 ICs. We evaluated our method for each data set type and find that results look very similar over all types with the clearest results for the conservative run. Following the classification scheme used in the study of Allen et al. (2011) it is plausible that the conservative data set gives the best and most valid results, since the ICs added in the intermediate and the liberal data set have worse spectral characteristics than the best artifact IC. As our method turned out to be stable concerning the three types of datasets, we show results of the conservative data set, since we want to deduce our results from data with the least amount of noise and RSNs having time courses with spectral characteristics at least better than artifactual ICs.

### 2.3. Multivariate empirical mode decomposition

After having decomposed our data set into a set of independent components consistent across a group of subjects, the next step then applied, for each session *i* separately, an MEMD on these time courses **T**_*i*_. MEMD represents a data-driven approach of decomposing e.g., non-stationary and non-linear time-series thus serving as a suitable tool for the analysis of brain data time courses. Following we present the concept of MEMD based on the canonical empirical mode decomposition (EMD) method. The approach was first introduced by Huang et al. (1998) and later extended to a noise-assisted ensemble EMD (EEMD) by Wu and Huang (2009). The decomposition results in IMFs, which represent characteristic inherent modes of the univariate time course under consideration. Let *x*(*t*) be a general univariate time-series, then EMD extracts one dimensional inherent modes *u*^*f*^(*t*) such that the original signal can be expanded into these modes plus a residual non-oscillating trend *r*(*t*) (Huang et al., 1998; Mandic et al., 2013)

(1) $$\begin{array}{r} x\left(t\right)=\overset{F}{\underset{f=1}{\sum }}u^{f}\left(t\right)+r\left(t\right) \end{array}$$

Note that EMD results in a complete decomposition of the signal, i.e., summing up all IMFs and the residue results in the original time course. Thus in contrast to exploratory signal decomposition techniques referred to above, absolute values of component amplitudes are of relevance giving each time point a unique partner over all IMF-indices. EMD starts by selecting all maxima and minima of *x*(*t*) and creates an envelope by spline interpolation for the sets of maxima and minima separately. Afterwards the mean of the envelopes is subtracted from *x*(*t*) and it is checked, if the resulting time course meets the criteria for being an IMF—this process is called *sifting*. The two criteria for a time course for being an IMF are: having symmetrical upper and lower envelopes and the number of extrema and zero-crossing differing at most by one (Mandic et al., 2013; Wang et al., 2010). If it does not meet the criteria, the process is started again with this new time course. Having extracted one IMF leads to the subtraction of the latter from the original time course, and the sifting process starts again with the remaining time course until a non-oscillating function is left, which is considered the residue *r*(*t*).

Mandic et al. (2013) mention at least two popular shortcomings of plain EMD. First, it is not ensured that modes appear in just one IMF, rather they could spread over several IMFs. This condition is known as *mode mixing*. Second, they mention a problem with so-called *end effect artifacts*, indicating that creating proper envelopes needs a sufficient number of extrema. As time courses, being decomposed, have finite range, the density of extrema tends to decrease near the edges of the sampled time interval. Therefore the fit of the envelopes is more error-prone at the beginning and end of the time courses.

**Figure 2:**
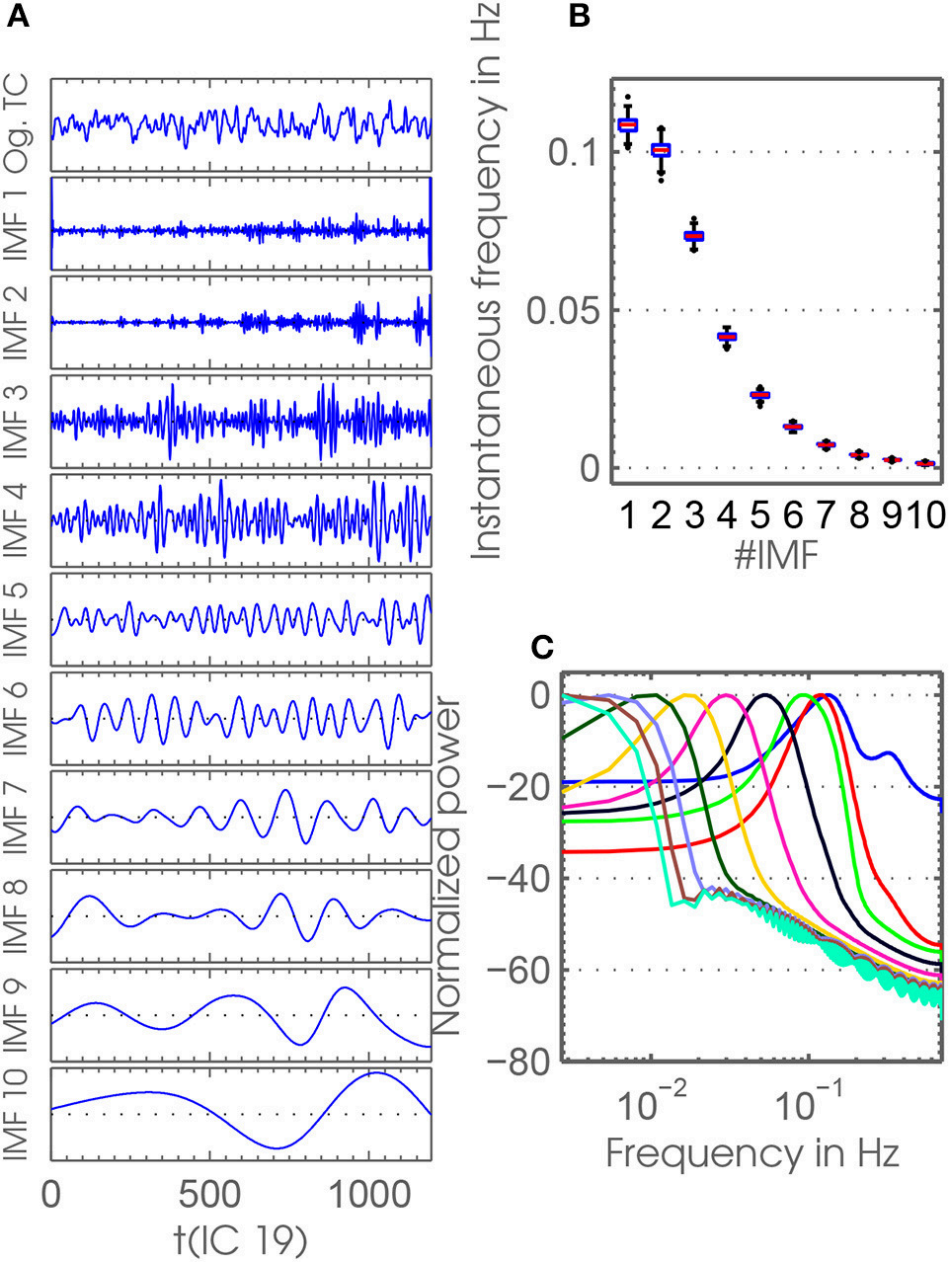
**(A)** The original time course and time courses of IMFs 1−10 of the first IC of one session (session 322) are shown as an example. **(B)** This panel shows box-plots for the instantaneous frequency against IMF indices. Data points for calculating the box-plots represent the instantaneous frequency averaged over time points and ICs. Black dots represent outliers concerning box-plots. MEMD mode alignment yields very small deviations in frequency between sessions, which enables us to do dFC on different frequency scales. **(C)** Depicted here are the power spectrum densities (normalized) for all IMF indices used. Each curve represents the average power spectrum densities over all ICs, sessions and subjects.

A further improvement of canonical EMD is provided by its extension toward a multivariate, noise-assisted ensemble EMD (MEMD) (Rehman and Mandic, 2009, 2011; Mandic et al., 2013). In short, EMD is extended to interpret input of multiple channels as a multi-variate signal. Compared to the noise-assisted variant EEMD, noise-channels are added to the multi-dimensional signal in the MEMD case instead of adding noise onto the time courses themselves. Creating an ensemble of IMFs by re-doing MEMD with newly generated noise as several realizations yields the noise-assisted ensemble extension of MEMD. Strictly speaking, the IMFs **U**^*f*^ extracted by MEMD are multivariate with the same dimensionality as the multivariate signals themselves. After creating an ensemble, representatives of IMFs are created by averaging over all realizations thereby reducing the mentioned shortcomings common to plain EMD (Rehman et al., 2013). One of the outstanding features of MEMD is its accurate mode-alignment quality, which renders it most suitable for a frdFC analysis. This is because for applying a sliding-window approach on separate frequency scales over many subjects and RSNs, we assume that the frequencies need to be aligned accurately. Therefore we use the MEMD approach for the decomposition of the time courses **T**_*i*_ of our data set resulting from the back-reconstruction step. MEMD is applied to **T**_*i*_ of each session. Figure 2A illustrates, as an example, time courses of the first IC from 10 IMFs of one session. The instantaneous frequencies of the modes decrease with increasing IMF-index. The box-plots of the frequency range of the IMF-indices (Figure 2B) and the corresponding spectral power densities (Figure 2C) show that mode mixing was avoided by separating frequencies of different scales. In particular, Figure 2B shows the distinct quality of MEMD to align modes over sessions and RSNs. This quality is also confirmed by low correlation values of IMFs between different indices (see Figure S2).

Before performing frdFC introduced below and applying MEMD on the time courses of the RSN ICs **T**_*i*_, a low-pass filter with a cut-off of 0.15Hz, despiking, and cubic detrending was applied on the time courses of the RSN ICs. This means that the time courses had the same level of preprocessing both before entering dFC, and MEMD. On the preprocessed time courses, we applied MEMD by using scripts provided by Rehman and Mandic (2009) (http://www.commsp.ee.ic.ac.uk/~mandic/research/emd.htm) after adapting them to the noise-assisted ensemble approach by adding four white Gaussian noise channels with a power of 6% (Rehman et al., 2013) of the average power of the original signal to the 24 time courses **T**_*i*_ of the RSN ICs in 30 realizations of noise for all 400 sessions. These noise channels were then discarded from all IMFs. For our further analysis, we used the first 10 IMFs only and discarded IMFs with higher indices. In some sessions and certain realizations, the algorithm stopped after IMF-index 10, which means that IMF 10 represented the residual signal. To assure comparability, we repeatedly initialized the algorithm with newly generated noise channels until it extracted at least 11 IMFs (10 IMFs + 1 residue), since this number was most common across all sessions and realizations. With this procedure we ensured the same stopping criterion throughout all sessions and that no residue was taken into account in calculations with time courses of IMF index 10. This procedure results in frequency-resolved time courses, or rather multivariate IMFs ${\text{U}}_{i}^{f}$ for each session *i* on frequency scales *f* = 1, …, *F* with *F* = 10.

### 2.4. Dynamic functional connectivity

For our analysis we needed to evaluate the dFC approach on the single-subject time courses of our data set **T**_*i*_ and on their corresponding frequency-resolved time courses ${\text{U}}_{i}^{f}$ (Figure 1B). For the whole study, the following parameters concerning dFC with constant window size hold. We use a boxcar window size of 80 TRs for the sliding-window approach resulting in a window length of 57.6 s. The time steps between each window are chosen to be 1 TR resulting in 1115 time steps for each session. Correlation matrices for each window were calculated using L1 regularization with 10 repetitions estimating precision matrices (Varoquaux et al., 2010; Smith et al., 2011) by applying graphical LASSO (Friedman et al., 2008). We ended up with sets of correlation matrices ${\text{X}}_{i}^{S}\left(t\right)$ from the standard time courses **T**_*i*_ and sets of correlation matrices ${\text{X}}_{i}^{I_{f}}\left(t\right)$ from time courses ${\text{U}}_{i}^{f}$ of IMF index *f* for each session. Every set of correlation matrices from one session had the dimension *C*×*C*×*T*, with *C* = 24 being the number of used RSNs and *T* = 1115 the number of windows. To stabilize variance, we applied a Fisher-transformation on every correlation coefficient before *k*-means clustering was performed. The *k*-means algorithm was applied to the set of correlation matrices from the original time courses ${\left\{{\text{X}}_{i}^{S}\left(t\right)\right\}}_{i=1,\ldots ,n}$ and the sets of correlation matrices from the time courses resulting from the MEMD ${\left\{{\text{X}}_{i}^{I_{f}}\left(t\right)\right\}}_{i=1,\ldots ,n}$ on each frequency scale separately with a maximum of 200 iterations and *k*∈[2;10]. These sets of correlation matrices that entered the clustering consisted of correlation matrices from all sessions. From each of the 10 frequency scales, and from the original time courses, 400 × 1115 correlation matrices resulted by applying dFC. On each of those sets of correlation matrices, *k*-means clustering was applied with *k* ranging from 2 to 10. Afterwards, Fisher-transformed correlation coefficients were back-transformed to original values for our analyses. Since *k*-means clustering depends on the initial conditions of the seed centroids, we initialized the algorithm several times with varying initial conditions. For this purpose, the *k*-means++ algorithm (Arthur et al., 2007) was applied before each *k*-means clustering run to find initial seeds. For all cases where rs-fMRI data was used [including null-models (section 3.3) and frequency dependent window size investigations (section 3.4)], the clustering was initialized 10 times.

We grouped our RSNs with respect to their similarity in the average correlation matrix (see arrangement in Figure 1A, left). This yielded an auditory/sensorimotor (AUD/SM), a visual (VIS), a cognitive control/visuospatial planning (CC/VSP), and a default mode/attentional module (DM/AT). So dividing our RSNs resulted in a clear modularization of the connectome with common functional properties of the members and distinct connectivity characteristics between different modules.

## 3. Results

The introduced pipeline enabled us to extract connectivity-states on different frequency scales. We investigated the scale invariance behavior of connectivity-states and found that there is a considerable drop of scale stability, when more than four connectivity-states have been extracted from rs-fMRI data by means of clustering. This finding has been corroborated by theoretical considerations and null-models. Additionally, we investigated simulated connectivity data with a known ground truth of different numbers of data inherent connectivity-states. By applying our proposed method on this data we found that exploiting scale invariance of connectivity-states yields a novel method of selecting the data inherent number of connectivity-states, or rather clusters. In *post hoc* filter bank investigations, we justified our use of MEMD by finding that using filter banks to enter frdFC depends on a delicate tuning of the underlying filters.

### 3.1. Robustness of connectivity-states over a wide frequency range

As introduced above, the dFC-analysis applied to the inherent time courses offered by the MEMD resulted in corresponding sets of inherent correlation matrices. We investigated the dynamics of connectivity-states, identified as centroids of a *k*-means clustering procedure, like in a standard dFC analysis. But, due to an MEMD preprocessing, we could analyze the dynamics on different inherent time scales as represented by the local frequencies and as identified through the IMFs extracted from the signals. To investigate the robustness of the connectivity-states across different inherent frequency scales as identified by MEMD, we employed the so-called Hungarian method (Kuhn, 1955; Munkres, 1957) to line up, across different frequency scales, most similar connectivity-states.

**Figure 3:**
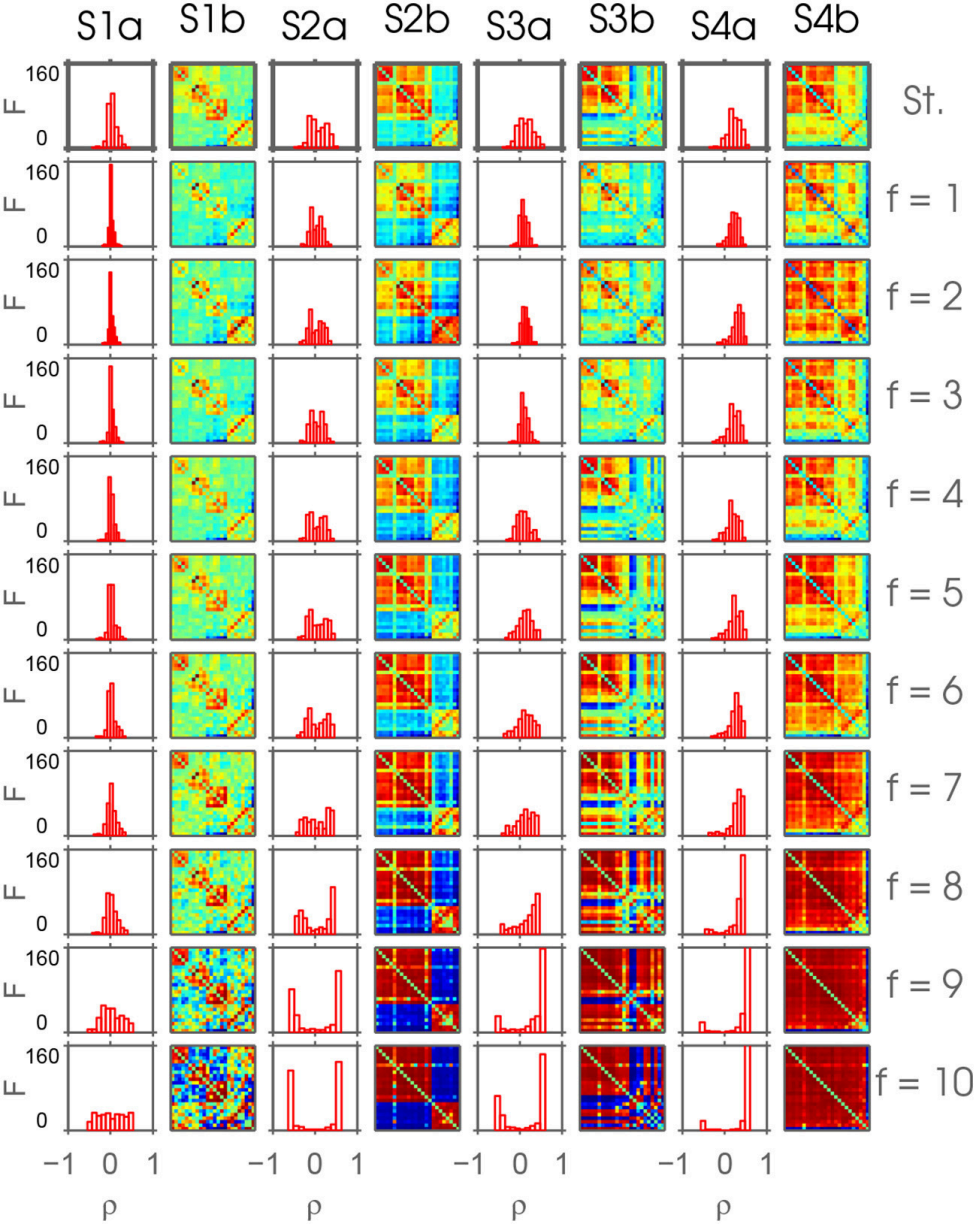
This figure depicts the result of the ordering Algorithm 1 applied on connectivity-states resulting from frdFC procedure using ${\text{U}}_{i}^{f}$ extracted by MEMD. This is a realization of the ordering procedure without shuffling the frequency scales *f* and *k* = 4 extracted connectivity-states on each frequency scale. In the top row, the connectivity-states resulting from the standard (St.) dFC procedure are shown. Below, each row shows the connectivity-states from frequency scales *f* = 1, …, 10 with increasing frequency defined by the IMFs resulting from MEMD. Each column S*b represents one connectivity-state, if it could be found robustly over frequency scales. Color represents the range from minimum (blue) to maximum (red) value of correlation to highlight the common structure over scales. The columns S*a show histograms of the absolute frequency of the correlation coefficients of the corresponding connectivity-states.

**Figure 4:**
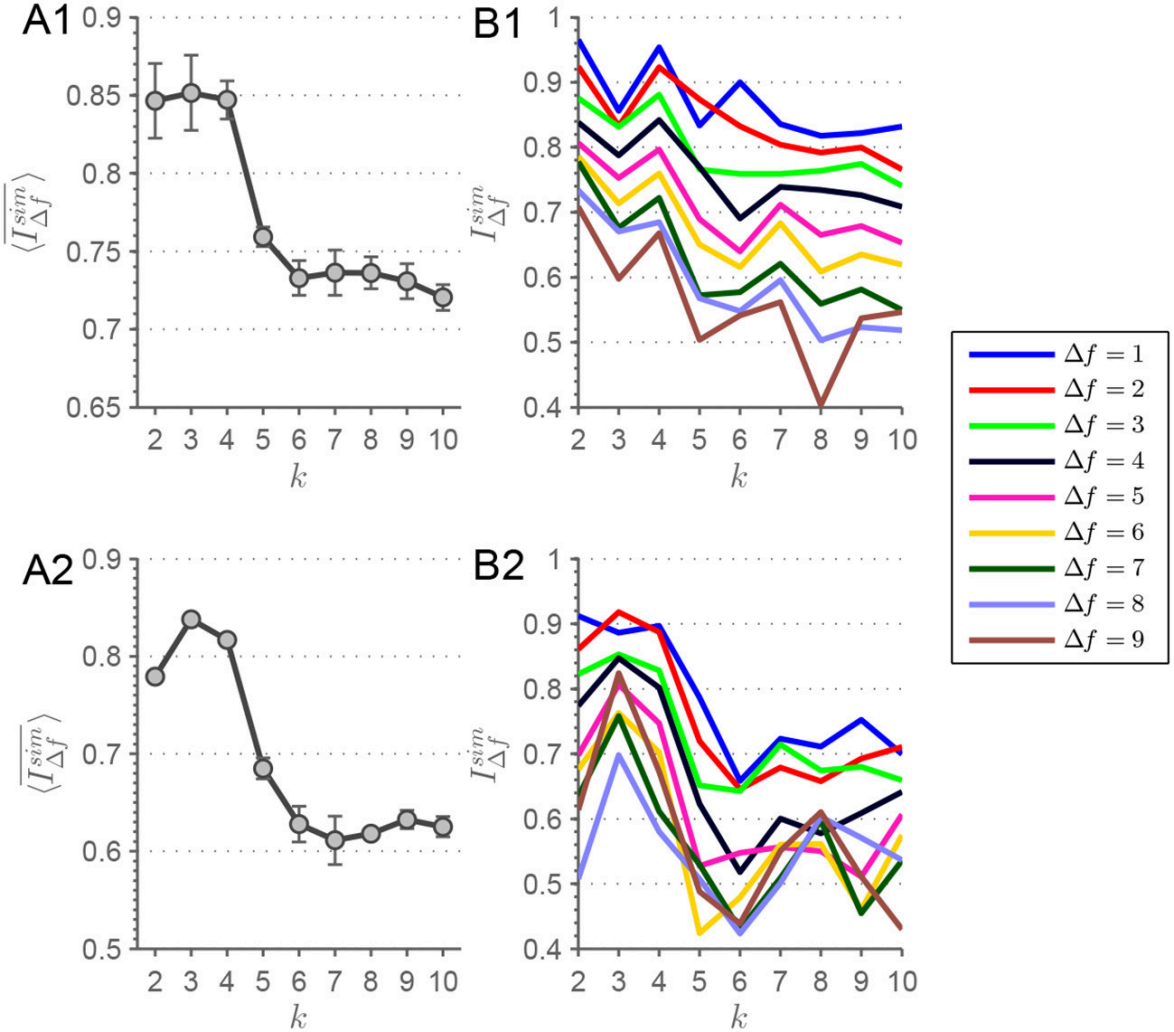
**(A1)** Here, the evolution of $\langle \bar{I_{\Delta f}^{sim}}\rangle \left(k\right)$ is depicted for the MEMD procedure. A clear drop off is visible from *k* = 4 to *k* = 5 extracted connectivity-states with a constant $\langle \bar{I_{\Delta f}^{sim}}\rangle \left(k\right)$ beforehand. In **(B1)**, $I_{\Delta f}^{sim}\left(k\right)$ is depicted for each Δ*f* from which $\langle \bar{I_{\Delta f}^{sim}}\rangle \left(k\right)$ of **(A1)** is derived. Analogously for **(A2,B2)**, but with the alteration of demeaning the connectivity-state matrices before applying the clustering procedure. Error bars depict the standard deviation. Lines are guide to the eye.

Applying *k*-means clustering separately to each set of correlation matrices of intrinsic modes with their related inherent frequency scales, results in an array of plots of dimension *F*×*k* (see e.g., Figure 3) with *F* extracted IMFs and *k* states according to the number *k* of underlying centroids. With varying *k*∈[2;10], this procedure resulted in nine plot arrays. The Hungarian algorithm sorts connectivity-states across all frequency scales according to closest similarity (see Figure 4 and Figures S3–S10 in the Supplementary Material). The Hungarian algorithm matched the connectivity-states ${\text{X}}_{j}^{{\text{I}}_{f+1}}$ of scale *f*+1 and column *j* to the average connectivity-state matrix ${\bar{\text{X}}}_{j}^{I_{1\ldots f}}$ over the preceding 1, …, *f* scales in column *j*. This is done for all *F* scales and then the configuration is saved. The averaging procedure of the first 1, …, *f* scales in each column *j* introduces statistical dependencies. Furthermore, the assignment procedure also depends on the configuration of scales. To account for these two aspects, the algorithm shuffles frequency scales before assignment starts using only non-repeating configurations. For each shuffling run *nIter*, the configuration after assignment is saved yielding *numIter* = 500 ordered configurations of *F*×*k* connectivity-states for further analysis. If *F*! < 500, then all possible permutations *numIter* = *F*! are used. Shuffling and averaging are introduced to the ordering procedure to avoid any sequence effects. The ordering procedure is summarized in Algorithm 1. For visualization purposes and a qualitative analysis, we illustrate the results of the realization with ordered frequency scales. In other words, for visualization purposes we show one particular *k*-means realization with no shuffling of frequency scales.

**Algorithm 1:** Ordering states over different frequency scales

Input: $\left\{{\text{X}}_{j}^{{\text{I}}_{f}}\right\}$ state matrices of scales *f* = 1, …, *F* and columns *j* = 1, …, *k*
1: for nIter = 1 : numIter **do**
2: Shuffle scales
3: for f = 1 : F - 1 **do**
4: Assign the states ${\text{X}}_{j}^{{\text{I}}_{f+1}}$ from scale *f*+1 to the average of the states ${\bar{\text{X}}}_{j}^{I_{1\ldots f}}$ of the preceding scales by employing the Hungarian method
5: end **for**
6: After states of the final scale have been aligned, save configuration and go to step 1)
7: end **for**

With *k* = 2, mainly results on lower frequency scales differ strongly from results on higher scales. Extracting *k* = 3 and *k* = 4 connectivity-states, respectively, (see Figure S3 and Figure 3) reveals a very robust alignment across all frequency scales. Note that with *k* = 4 connectivity-states, another robustly aligned column, i.e., state, is added to the three connectivity-states already obtained from the run with *k* = 3. For larger number *k* of clusters, this is no longer the case, since newly added columns become increasingly misaligned reflecting a larger diversity among connectivity-states.

Visual inspection of the aligned connectivity-states is already encouraging. In Figure S3, the result of extracting *k* = 2 connectivity-states from different frequency scales resulting from MEMD and subsequent ordering by applying Algorithm 1 is depicted. State S1b is robust at least up to scale *f* = 8 and S2b up to scale *f* = 7, but with more pronounced heterogeneity. For *k* = 3 in Figure S3, the first two states resemble the same states found for *k* = 2. Additionally, a third connectivity state (S3b) shows up. In this array, all three states show high robustness over scales. In the following, I refer to this robustness of states over scales as scale stability. Also for *k* = 4 (Figure 3) a new state (S4b) shows up being robust over scales, with scale stability of the other three states still being preserved. When extracting one more connectivity-state from each scale, this pattern of scale stability breaks down. In Figure S5, a vast loss in scale stability can be observed. Three connectivity-states still show robust scale stability, which is lost for the connectivity-state found in the first column of Figure 3. This loss in scale stability is also preserved when extracting *k*>5 connectivity-states (see Figures S6–S10). The tendency of increasing correlation coefficients evident in the histograms shown in each of those figures, can be explained by the increasing period of the time courses and the constant window size used for the sliding window approach.

### 3.2. Variability of scale-stability of connectivity-states

Looking at connectivity-states across frequency scales more quantitatively reveals that connectivity-states, deduced from particular *k*-means groupings, are highly stable concerning their similarity. The similarity of states can be looked at more closely by correlating connectivity-states across adjacent frequency scales. After aligning connectivity-states for each *k*-means run, patterns at frequency scales *f* and *f*+1 are correlated – thus Δ*f* = 1 in the sense of frequency “distance.” Then Δ*f* is increased step by step to look for similarity at more distant frequency scales. Finally, the global measure of similarity is plotted for each Δ*f* (see Figure 4B1).

Correlation is determined by looking for highest association between patterns. The latter is estimated by averaging all correlation coefficients resulting from the comparison of states across all frequency “distances.” By doing so we achieve a measure of similarity for each *k* and each Δ*f* according to

(2) $$I_{\Delta f}^{sim}(k)=\frac{1}{(F-\Delta f)k}\sum_{i=1}^{k}\sum_{f=1}^{F-\Delta f}\text{corr}\left(X_{i}^{f},X_{i}^{f+\Delta f}\right).$$

Averaging $I_{\Delta f}^{sim}$ over Δ*f* results in a global similarity measure $\bar{I_{\Delta f}^{sim}}$ for a complete *k*-means run, i.e., for each *k*. This measure takes into account both similarity over nearby as well as distant frequency scales. To avoid sequence artifacts or any weighting of scales, the ordering algorithm shuffles frequency scales before ordering. Each shuffling iteration, the scale-stability index of Equation 2 is calculated and afterwards averaged over the ensemble. The *k*-means clustering procedures are initialized ten times with varying initial seeds. This means, for each of those realizations $\langle \bar{I_{\Delta f}^{sim}}\rangle \left(k\right)$ is calculated and its evolution with *k* can be plotted including error bars representing the consistency over all those realizations. Plotting this ensemble average $\langle \bar{I_{\Delta f}^{sim}}\rangle \left(k\right)$ for each *k*, results in a high similarity across frequency scales of connectivity-states for *k* ≤ 4, but similarity decreases for larger numbers of extracted states corresponding to *k*∈[5;10] (Figure 4A1). This drop of $\langle \bar{I_{\Delta f}^{sim}}\rangle \left(k\right)$ from *k* = 4 to *k* = 5 can be seen by looking at Figure S5.

In a recent study, Leonardi et al. (2014) investigated dFC and connectivity-states by suggesting that dFC matrices should be demeaned before entering the clustering procedure resulting in better clustering. By demeaning they understand to subtract the temporal mean of each correlation function *X*_*ij*_(*t*) from its time course, i.e. each entry of the dFC matrices is temporally demeaned. In Figure 4A2, the scale stability measure $\langle \bar{I_{\Delta f}^{sim}}\rangle \left(k\right)$ is shown for this type of procedure applied to the data used in this study. The demeaning procedure is applied after correlation coefficients have been Fisher transformed. This results in a peak at *k* = 3, but with a sharp drop off evident from *k* = 4 to *k* = 5. Looking at the *F*×*k* array for *k* = 4 of the demeaned data (Figure S11), it can be seen that the state resembling static functional connectivity is absent, and the other three states are similar to the remaining states from Figure 3. Also the plots of $I_{\Delta f}^{sim}\left(k\right)$ are shown in Figure 4B2 for the demeaned data.

Theoretical considerations show that the pattern depicted in Figure 4A1 can also be mathematically deduced (see section 1.5 in the Supplementary Material).

### 3.3. Null-model tests

**Figure 5:**
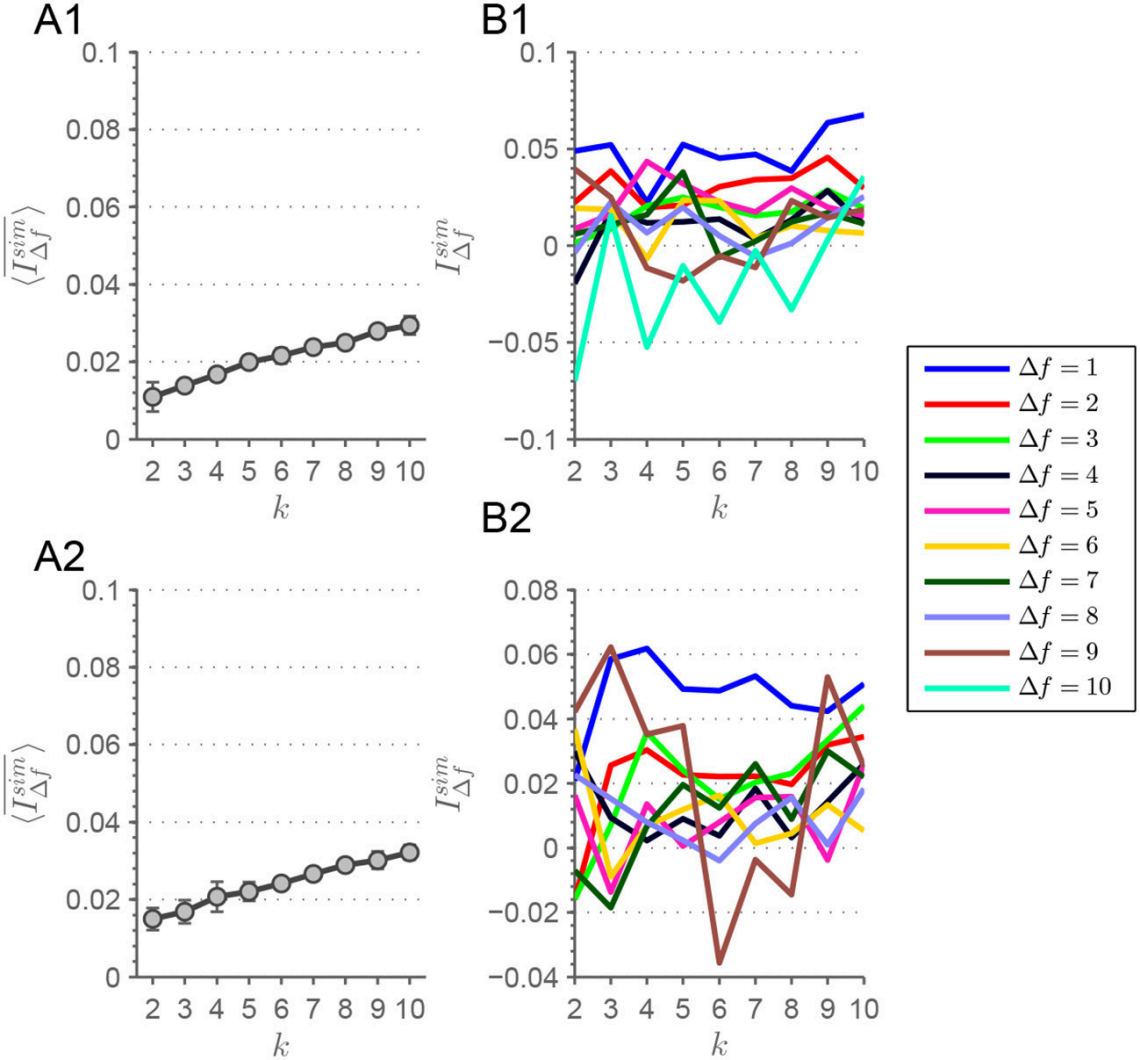
**(A1)** Depicts $\langle \bar{I_{\Delta f}^{sim}}\rangle \left(k\right)$ from the shuffled and **(A2)** from the phase-randomized time courses. **(B1,2)** Show $I_{\Delta f}^{sim}$, from which $\langle \bar{I_{\Delta f}^{sim}}\rangle \left(k\right)$ is derived, for the corresponding time courses. Error bars depict the standard deviation. The lines are guides to the eye.

We tested the validity of our results by applying our approach to two null-models derived from the original **T**_*i*_. Time courses from both null-models entered the pipeline at the same level as the original time courses **T**_*i*_ (example plots of the clustering can be found in Figures S13, S14). The first null-model was introduced by shuffling the standard time courses **T**_*i*_ for each session *i* and each RSN *c* = 1, …, *C*, which destroys any temporal structure present in the data. The second null-model was derived from phase-randomizing each RSN time course from **T**_*i*_. This null-model ensures that a frequency relevant structure is preserved in the time courses while destroying the covariance patterns (Prichard and Theiler, 1994; Allen et al., 2014). An MEMD yielded more IMFs (Figure S13) for the shuffled time courses, since shuffling introduces high frequencies not present beforehand. The results of the null-model tests are shown in Figures 5A1,2,B1,2. It can be seen that both approaches with surrogate time-series result in an almost vanishing scale-stability index or even show an opposing trend with slightly increasing stability with increasing *k* (Figures 5A1, 2).

### 3.4. Frequency dependent window size

**Figure 6:**
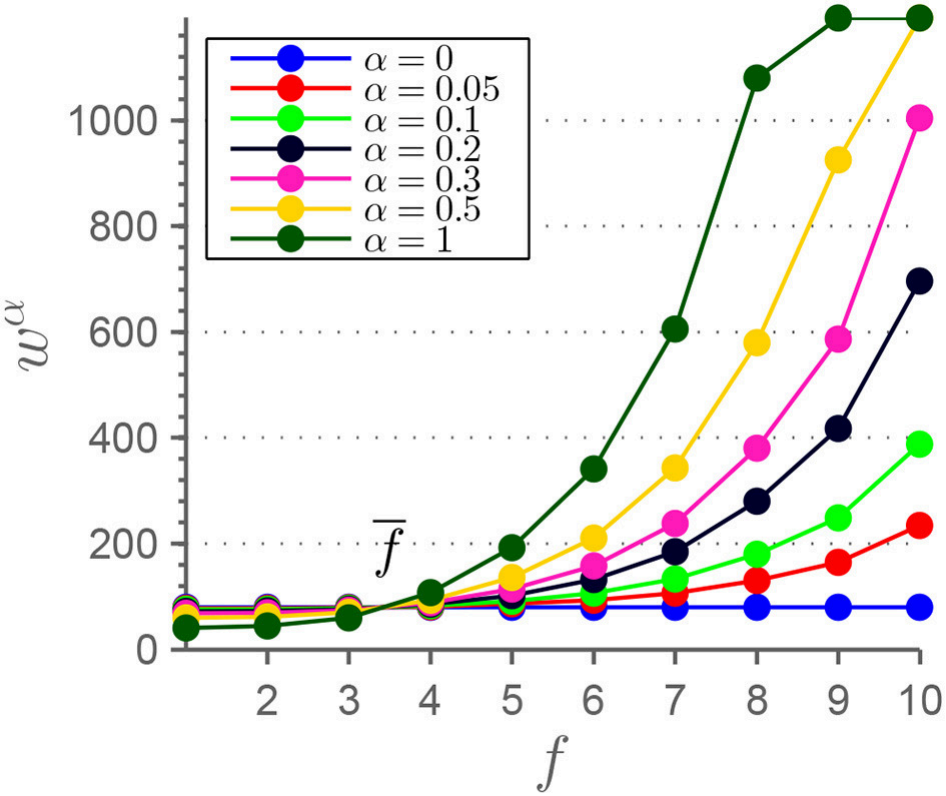
This figure shows the adjustment of window size *w*^α^ on frequency scales *f* used for the sliding-window approach. The α-values range from the one extreme case with constant window size (α = 0) to the case with a constant average number of periods in each window (α = 1).

We also investigated the dependence of the window size used in the sliding-window approach. The above reported results are extracted using a constant window size of $w\left(\bar{f}\right)=57.6$ s. This size is applied both to the frequency-resolved time courses resulting from MEMD, and the original time courses. To provide a reference of how many periods are covered by this window size in the original time courses, we calculated the fast Fourier transformation of the average spectrum over all used RSNs and sessions. The weighted-average frequency is $\bar{f}\approx 0.055$ Hz and the number of periods covered by a window with size $w\left(\bar{f}\right)$ is $\bar{n_{T}}\approx 3.16$. To adjust the window size to the average instantaneous frequency *f* of the IMFs, we added

(3) $$\begin{array}{r} \Delta w^{\alpha }\left(f\right)=w\left(\bar{f}\right)\left(\frac{\bar{f}}{f}-1\right)\cdot \alpha \end{array}$$

to $w\left(\bar{f}\right)$, resulting in an adjusted, frequency dependent window size *w*^α^(*f*). We chose to use α∈{0.05, 0.1, 0.2, 0.3, 0.5, 1} resulting in *w*^α^(*f*) as plotted in Figure 6. For α = 0.5∧*f* = *f*_9_ and α∈{0.5, 1}∧*f* = *f*_10_ *w*^α^(*f*) exceeded *T*. In those extreme cases, window size was chosen to be *w*^α^(*f*) = *T*−1. For α = 1, the frequency dependence resembles the case where the number of periods in a window is equal across all frequency scales (except the mentioned cases)

(4) $$\begin{array}{r} w^{1}\left(f\right)=w\left(\bar{f}\right)\cdot \frac{\bar{f}}{f}. \end{array}$$

**Figure 7:**
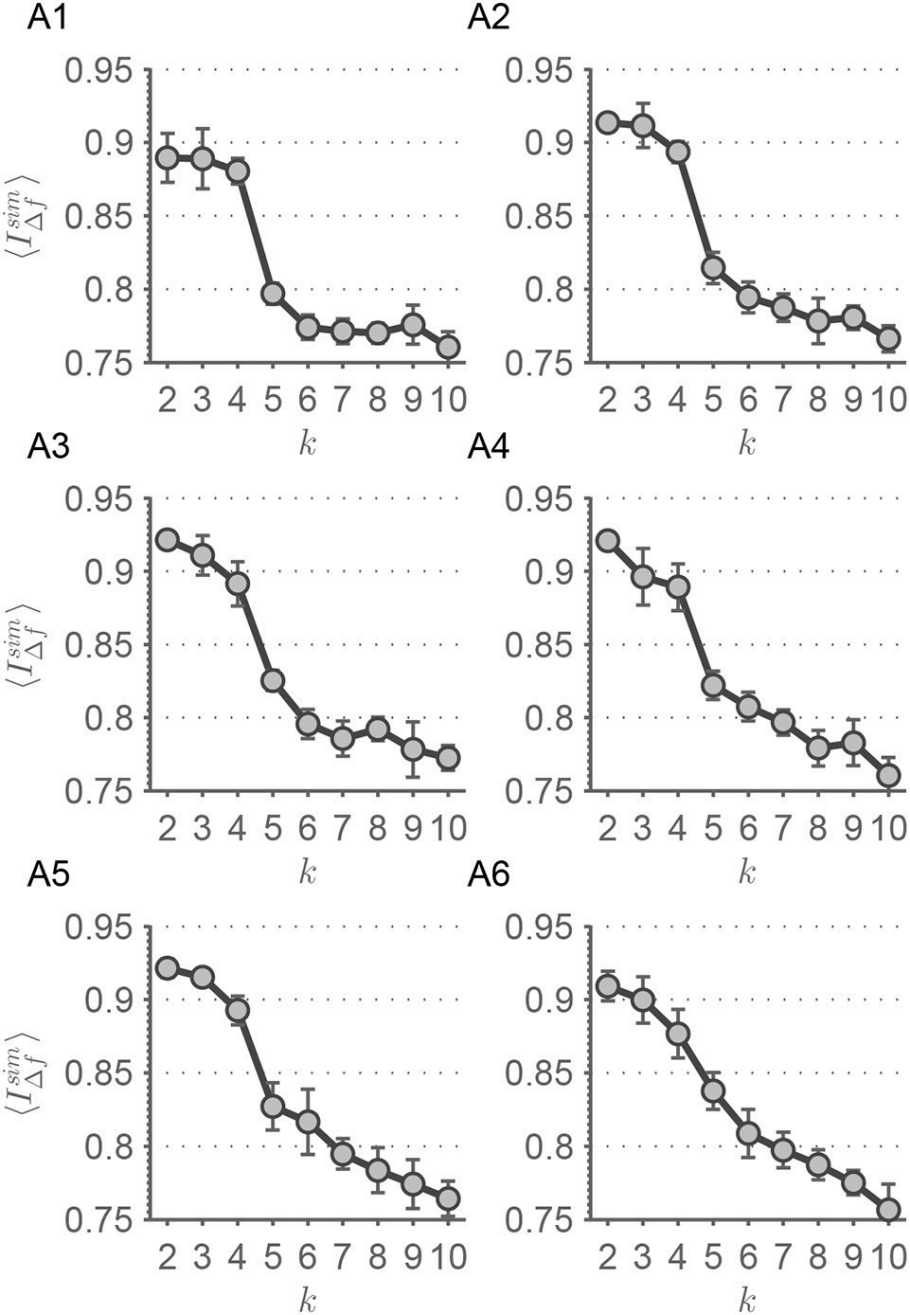
In this figure, the results from the frequency dependent window size procedure are shown. Each panel represents the evolution of the scale stability measure $\langle \bar{I_{\Delta f}^{sim}}\rangle \left(k\right)$ for each value of α∈{0.05, 0.1, 0.2, 0.3, 0.5, 1}. **(A1)** α = 0.05, **(A2)** α = 0.1, **(A3)** α = 0.2, **(A4)** α = 0.3, **(A5)** α = 0.5, **(A6)** α = 1. Error bars represent the standard deviation of $\langle \bar{I_{\Delta f}^{sim}}\rangle \left(k\right)$. The lines are guides to the eye.

Afterwards, the sliding window approach was conducted with window size adapted to different frequency scales. For each α-value and each frequency scale *f*, *k*-means clustering was applied in analogy to our introduced procedure, which would be represented by α = 0. For each α-value, $\langle \bar{I_{\Delta f}^{sim}}\rangle \left(k\right)$ was calculated and plotted against *k* (Figure 7). Also with variable window size a comparable pattern emerges with larger drop-offs from *k* = 4 to *k* = 5.

### 3.5. Simulated dynamic functional connectivity

**Figure 8:**
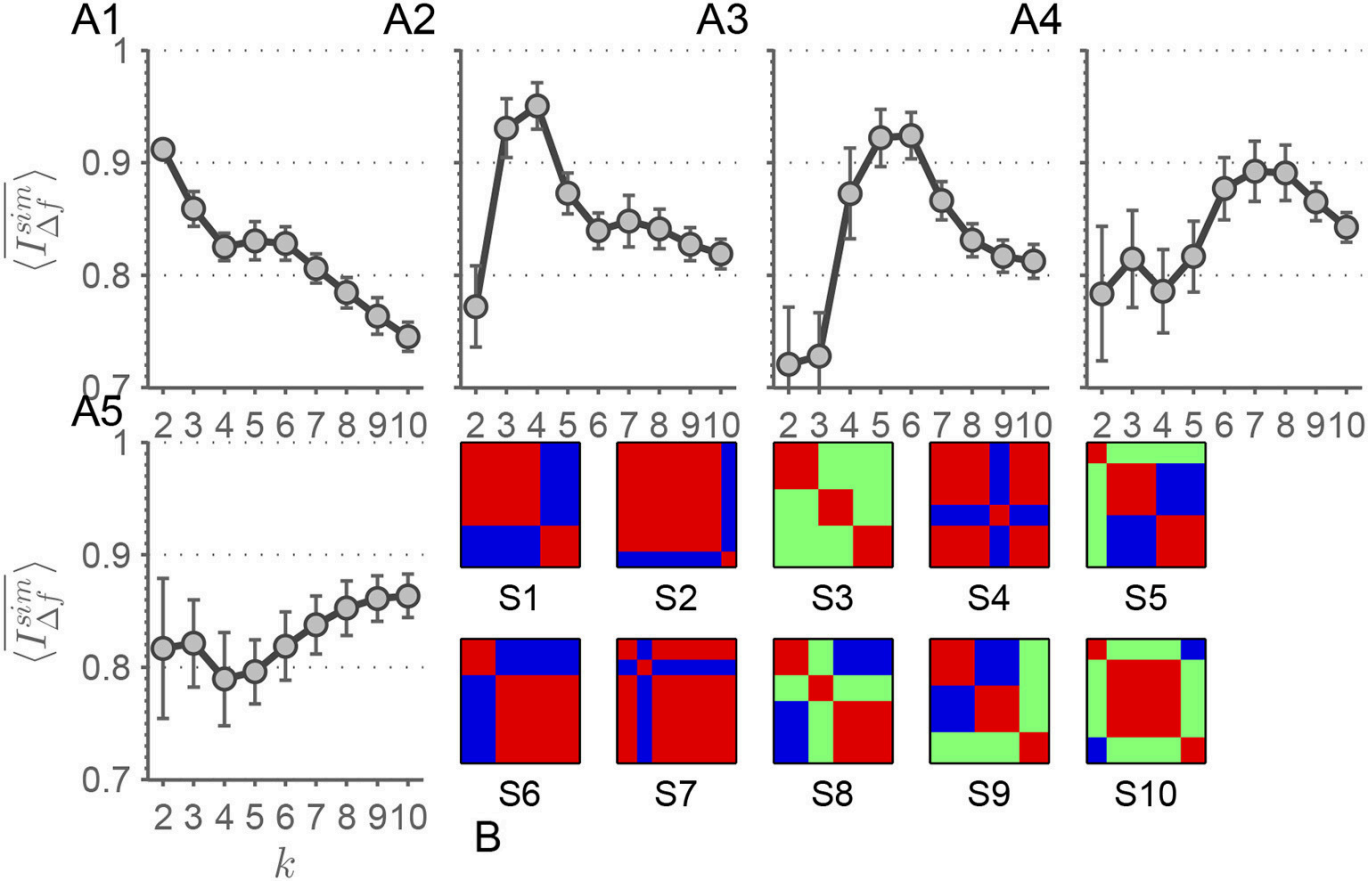
**(A1–5)** In these panels, selected results of $\langle \bar{I_{\Delta f}^{sim}}\rangle \left(k\right)$ are shown for *k*_*sim*_ = 2 **(A1)**, *k*_*sim*_ = 4 **(A2)**, *k*_*sim*_ = 6 **(A3)**, *k*_*sim*_ = 8 **(A4)**, and *k*_*sim*_ = 10 **(A5)** simulated connectivity-states. Error bars depict the standard deviation. **(B)** depicts the artificial states **(S1–10)** the simulated rs-fMRI time-series traverse. Each correlation matrix has the dimension 24 × 24. Blue color represents anti-correlation, red correlation, and green no correlation.

In order to validate our approach and strengthen our results, we applied our method to simulated data from the toolbox *SimTB* (Erhardt et al., 2012), which enables us to simulate dFC traversing a predefined number of connectivity-states *k*_*sim*_. To do that we modified the script offered in the course of the study of Allen et al. (2014) (http://mialab.mrn.org/software/simtb/docs/create_toysimulation.m). We simulated 24 RSN time courses with a length of *M* = 1, 200 time points and a TR = 0.7 s. The number of simulated subjects and sessions was chosen so that in each simulation run with a particular number of artificial connectivity-states the number of occurrences of each state was *n*_*occ*._ = 96. The sequence of connectivity-states was randomized, but with the constraint that consecutive intervals always traverse different connectivity-states. Thus, the transition between states was randomized. The duration of each connectivity-state was chosen to be Δ*T* = 150 TRs. We chose the numbers of connectivity-states to be simulated as *k*_*sim*_∈{2, 4, 6, 8, 10}, the prototypes of which can be found in Figure 8B. There are three main parameters that can be adjusted—the probability of a unique event *p*_*u*_ and its amplitude *a*_*u*_ in relation to the amplitude of the coherent, or rather, state event with the occurence probability *p*_*u*_. We probed this three dimensional parameter space, but an exhaustive parameter sweep was not possible due to the high computational demand. We simulated all possible combinations of the values {0.6, 0.8, 1}. We show parameter sets with lower values in the noise terms than in the signal term (Figure 8A1: *p*_*u*_ = 0.8, *a*_*u*_ = 0.8, *p*_*s*_ = 1; Figure 8A2: *p*_*u*_ = 0.8, *a*_*u*_ = 0.6, *p*_*s*_ = 0.8; Figure 8A3: *p*_*u*_ = 0.6, *a*_*u*_ = 0.8, *p*_*s*_ = 0.1; Figure 8A4: *p*_*u*_ = 0.8, *a*_*u*_ = 0.8, *p*_*s*_ = 1; Figure 8A5: *p*_*u*_ = 0.8, *a*_*u*_ = 0.6, *p*_*s*_ = 0.8) as a proof-of-principle, since most of the parameter combinations show comparable results. $\langle \bar{I_{\Delta f}^{sim}}\rangle \left(k\right)$ shows distinct patterns for each *k*_*sim*_∈{2, 4, 6, 8, 10} (Figures 8A1–5) with the tendency to peak at *k* = *k*_*sim*_.

### 3.6. Filter-banks

In a *post hoc* analysis we apply Butterworth filter-banks both on the rs-fMRI and simulated time courses. We find that a filter-bank emulating bands with similar bandwidths like the frequency scales resulting from MEMD cannot be realized in a sensible manner. The frequency scales represented by IMF indices *f*_7_ to *f*_10_ have a very narrow bandwidth, lie close together, and are represented by frequencies very close to zero (Figure 2B). Therefore, we switch to filter-banks with equidistant bands. All orders of the used filters are chosen to result in stable pole behavior. We followed a more canonical and a more adjusted way of constructing the filter-banks. In the canonical way, the order of the filters was chosen to be constant for filter-banks of a certain number of bands. Hence, we chose order 10 for *n*_*bands*_ = {5, 8} bands, order 8 for *n*_*bands*_ = {10, 12} bands, and order 6 for *n*_*bands*_ = 15 bands. In the adjusted way, we design the filters with the maximum possible order barely resulting in stable filter behavior adjusted for each band in each filter-bank. This results in varying filter orders over different bands. The two filter-bank designs can also be considered as a conservative and a liberal design, respectively. For both approaches, the lowest bands are realized as low-pass filters. Since the preprocessing of the rs-fMRI time courses includes a low-pass filter with a cut-off frequency of 0.15 Hz, we partition the interval [0 Hz; 0.15 Hz] in bands each with bandwidth $\Delta \nu =\frac{0.15Hz}{n_{bands}}$. We evaluate our approach on the resulting time-series on *n*_*bands*_ different frequency scales.

**Figure 9:**
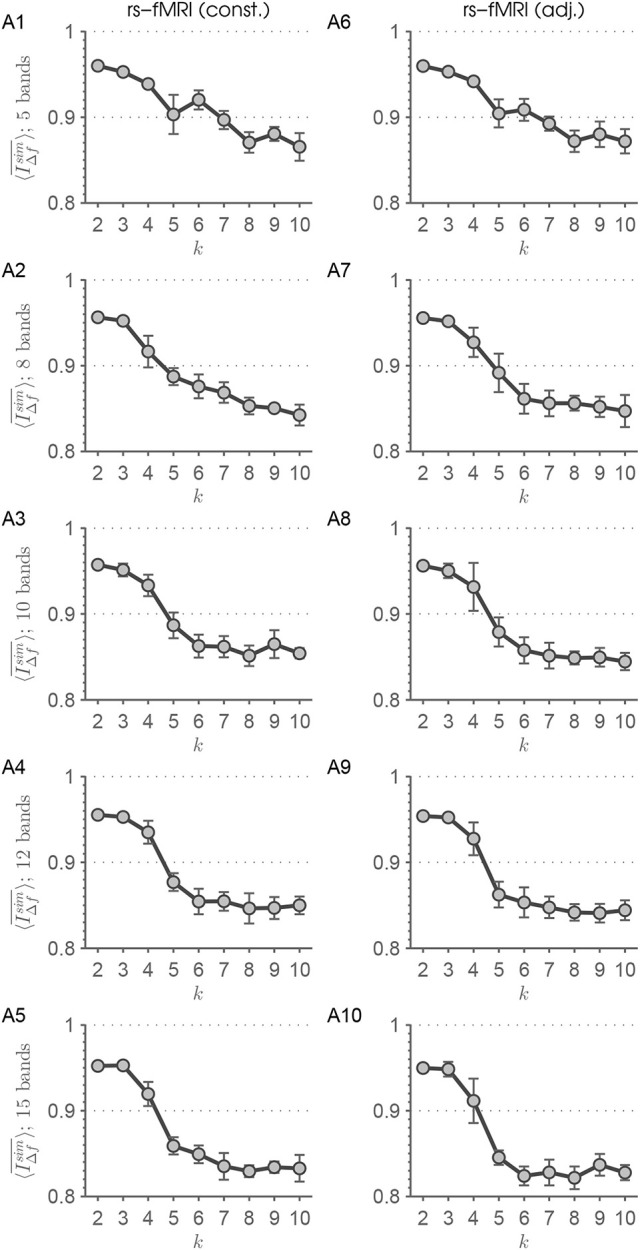
This figure summarizes the results of the scale-stability analysis for the filter-bank procedures with constant **(A1–5)** and adjusted filter order **(A6–10)** applied on rs-fMRI data. It can be seen that increasing the number of extracted frequency bands results in a clearer drop-off in scale stability of connectivity-states. Therefore, applying MEMD has the advantage of avoiding to delicately tuning the number of extracted frequency bands.

Figure 9 shows the results of this approach applied on rs-fMRI data. With increasing number of extracted frequency bands, the drop from *k* = 4 to *k* = 5 found by the MEMD approach (Figure 4) becomes more evident. For *F* = 5 and *F* = 8 there is almost no drop-off. Furthermore, differences between the constant and adjusted filter order approach are very subtle. Comparing Figures 9A3–5 with Figures 9A8–**10** shows a greater uncertainty in terms of larger standard deviations adjacent to the drop off for the adjusted order approach. This means, when taking the results found in Figure 4 as the desired outcome, Figure 9A5 shows the clearest result with least uncertainty. Figure S15 shows the results of this approach applied on simulated data with *k*_*inh*_ = 4 states. With increasing number of frequency scales the pattern identifying data inherent connectivity-states becomes clearer. Additionally, when using frequency scales extracted by filter banks, the scale stability measure $\langle \bar{I_{\Delta f}^{sim}}\rangle \left(k\right)$ shows values ≈1, which represents almost perfect stability of connectivity-state structure over scales.

### 3.7. Data repository

We provide a data repository of our frequency-resolved time-series.^2^ In this repository we include the time-series resulting from the MEMD procedure and filter-bank procedures both for constant and adjusted filter order and the MEMD decomposed time-series from the simulation runs plus the not shown simulations (all main effects and interactions). In addition to the decompositions we also offer the original, non-decomposed time courses with the full preprocessing level where applicable (not for simulations). The dFC matrices are stored on local servers and can be retrieved on demand.

## 4. Discussion

### 4.1. General statement

We applied MEMD to complete time courses of RSNs resulting from a gICA analysis, i.e., before the sliding-window procedure was employed to perform a dFC analysis. We showed that MEMD offers the possibility of comparing IMFs over sessions and RSNs, since it aligns the modes accurately. This results in time courses that can be compared within each IMF-level across all sessions and RSNs, opening up a way to perform a dFC analysis on different frequency scales. This suggested a frdFC analysis. After the application of a sliding window procedure on frequency-resolved time courses, clustering of the sets of correlation matrices has revealed scale stability as an inherent feature of connectivity-states, or rather cluster centroids. The dependence of scale-stability of connectivity-states on the number of extracted clusters is a novel finding and could be related to existing literature describing scale-invariance as a characteristic feature of rs-fMRI. Our approach introduces a novel method of inferring the number of data inherent connectivity-states. In *post hoc* analyses, null-model and filter bank investigations, as well as simulated data, our main findings have been corroborated.

When it comes to apply MEMD, time courses are decomposed into a locally orthogonal set of IMFs that – when summed up – result in the original time-series. Investigating frdFC by means of MEMD yields two conflicting considerations to be taken into account. On the one hand, since time courses are analyzed at different time scales and the related local frequencies are decreasing with increasing IMF index, adjusting window size by Equation 4 is an obvious adaptation of the approach. On the other hand, the representation of the original time course by the superposition of all IMFs prefers the case with constant window size where α = 0 in equation 3. This assures that, within any given time window, time samples of different modes correspond accurately to each other across all modes, i.e., each time point has its exact partner for each IMF index. As a consequence, choosing an adaptive window size yields correlation matrices which are less comparable across different frequency scales, since for modes with higher IMF-indices, the time window encompasses time samples which do not belong to corresponding time windows in modes with lower IMF-indices. However, choosing α = 0 neglects the increasing period of intrinsic local oscillations with increasing IMF index. Those two lines of thought have to be kept in mind when performing frdFC analysis by means of MEMD. Yet another consideration favors the approach with constant window size when it comes to apply *k*-means clustering. Adapting window size to the period of local oscillations in different modes also leads to varying numbers of data points in the clusters if *k*-means is applied at different frequency scales. In the extreme case with α = 1, *k*-means runs with *n*_*f*_1__ = 461, 600 and *n*_*f*_10__ = 400 data points are compared. Statistically, this severe imbalance offers the algorithm the possibility to extract a larger number of centroids for IMF index *f*_1_ and a vastly reduced space of possible centroids for *f*_10_. With these considerations in mind, the approach with constant window size seems to be preferable over the one with time scale adapted window sizes. Furthermore, the striking similarity of connectivity-states across frequency scales for *k* = 4 in the constant window case lends credit to prefer this approach over the adaptive one. Also the findings of the frequency adjusted window size investigations show much clearer results in terms of detectability of data inherent connectivity-states. But nevertheless, when considering both approaches equivalently, our results still consistently show a high $\langle \bar{I_{\Delta f}^{sim}}\rangle \left(k\right)$ for *k* ≤ 4 and above this value $\langle \bar{I_{\Delta f}^{sim}}\rangle \left(k\right)$ drops. The simulated dFC traversing artificial connectivity-states (Figure 8) strengthen the result of *k* = 4 inherent scale-invariant states in the data. The peak of $\langle \bar{I_{\Delta f}^{sim}}\rangle \left(k\right)$ is more pronounced for *k*_*sim*_ = 4 in the simulated case (Figure 8A2), which can be expected in such an idealization.

### 4.2. Scale-stability and its possible benefits for connectivity-state extraction

In the course of this study we also looked at elbow-criteria after *k*-means runs as a selection criterion for the proper number of extracted connectivity-states. Unfortunately, no clear elbow-criterion could ever be established. Using within-cluster similarity as a measure of choice, clear elbows are rarely evident. Therefore selection of a certain number of extracted clusters would have been always subjective. Moreover, employing Silhouette scores also resulted in an insufficiently pronounced clustering structure for the original time course case.

Regarding this fact, and our results concerning varying scale-stability of connectivity-states across frequency scales and model order *k*, we rather suggest to consider scale-stability $\langle \bar{I_{\Delta f}^{sim}}\rangle \left(k\right)$ as a proper measure to infer model order. The latter could be identified as the number of clusters/connectivity-states which show highest scale stability across the relevant frequency scales. Our study shows that *k* = 4 connectivity-states deem most appropriate for the data that has been analyzed. This optimization for scale-stability is in line with literature reports which suggest scale-invariance as an inherent feature of rs-fMRI. In Kitzbichler et al. (2009), the authors argue that phase-synchrony is an important feature for network formation at all frequencies in resting-state conditions. Since the extraction of connectivity-states from dynamically changing correlation matrices implies mutual coherence, a similar argument holds here. Thus this argument is also in favor of identifying model order with the number *k* of connectivity-states with highest scale-stability across all inherent frequency scales.

The main finding of a high level of stability for *k* ≤ 4 and the drop-off from *k* = 4 to *k* = 5 is robust over several modifications. Intriguingly the recently suggested component-wise temporal demeaning of dFC matrices (Leonardi et al., 2014) yields similar results in terms of stability for *k* ≤ 4. Leonardi et al. (2014) used a considerably smaller sample of sessions compared to our dataset. We suggest that component-wise temporal demeaning seems to be more crucial for studies with smaller cohorts than large population studies and that its effects average out with increased number of used sessions. We suppose that for smaller cohort studies demeaning seems to be a crucial aspect (Leonardi et al., 2014), but for large population studies the effect of demeaning seems to diminish in terms of feature extraction by means of *k*-means. Also the simulations conducted (Figure 8) point to a benefit in scale-stability considerations when it comes to inferring the underlying number of connectivity-states in a dataset. For each *k*_*sim*_ a distinct pattern in $\langle \bar{I_{\Delta f}^{sim}}\rangle \left(k\right)$ with the tendency of peaking at *k* = *k*_*sim*_ emerged. When considering the scale-stability findings of MEMD, filter-banks, simulated and rs-fMRI data together, then we have accumulated evidence that data-inherent connectivity-states emerge in a stable manner over several scales and that our approach is appropriate for determining the data inherent clustering structure.

### 4.3. Filter-banks, MEMD, and self-similarity

Analyzing expectably self-similar time-series by means of EMD is a natural way of approaching such data (Flandrin et al., 2004). Exploring the frequency structure in more detail via filter-banks revealed striking robustness of the scale-invariance feature concerning connectivity-states. Especially, in the case of simulated time courses cycling through *k*_*sim*_ = 4 artificial connectivity-states, where the scale-invariance of those states holds for different numbers of bands, i.e., different frequency resolutions. For rs-fMRI time-series the variation in the number of bands and with it the variation in bandwidth yields less robust results, especially in the fourth connectivity-state. For real data it seems that the design of the filter-bank is more crucial than for simulated data. Nevertheless, we conclude that real and even simulated rs-fMRI time-series cycling through connectivity-states imprint their coherence not only on very distinct frequencies, but rather on a much broader spectrum. MEMD in turn seems to be capable of finding bands in a data-driven manner that consist enough information to reveal the scale-invariance of connectivity-states. Nevertheless, also filter-banks posses this capability, but may be more sensitive to fine tuning parameters.

We want to stress the fact that the pattern in the simulated (Figure 8A2) and rs-fMRI data (Figure 4A1) are quite similar. This pattern can also be found in most of the parameter combinations of the simulated data. Comparing the filter-bank findings from the simulated data (Figure S15) with those two MEMD findings, the main pattern always holds and can be carved out when neatly designing the filter-bank. Considering the rs-fMRI data it seems that the design of the filter-bank has to be more precisely chosen than for artificial data, since the patterns shown in Figure 9 vary slightly depending on the chosen filter-bank. This finding is not surprising, since in real data more noise-types should be present in the time courses than can be simulated by the unique events introduced to the artificial time-series. Since, the finding of a scale-stability drop-off from *k* = 4 to *k* = 5 is this common in our studies, we consider it as a benchmark pattern concerning our used approaches and scale-definitions. Assuming this pattern being the desired outcome of the stability analysis, we find that the results from the filter-banks are susceptible to parameter tuning of the filter. Nevertheless, MEMD seems to be capable of finding the bands, which result in similar patterns in simulated and real data.

### 4.4. Limitations and future directions

The time scales of extracted IMFs have also to be discussed. Recent research showed that fluctuations in cortical activity have physiological foundations even in the infra-slow frequency range (Pan et al., 2013; Hiltunen et al., 2014). This means that intrinsic modes with indices up to *f*_7_ can still be of physiological origin. For even slower IMFs, corresponding to large periods or, equivalently, low instantaneous frequencies, a physiological interpretation is not immediately obvious and needs further investigations. In this respect it is worth mentioning that even on long (low) time (frequency) scales, the structure of the four connectivity-states, which we were able to identify, is still preserved. Hence, we are not dealing with artifacts on these time scales. Thus the question arises: why is it possible to literally define connectivity-states exploring the slowest fluctuations, which result from the decomposition of cortical activity and exploiting the limited information available from them? We claim that our findings corroborate self-similarity and scale-freeness, which indeed has been found in rs-fMRI (Eguíluz et al., 2005; Kitzbichler et al., 2009; Fraiman et al., 2009; Tagliazucchi et al., 2012), which demonstrates the emergence of similar structures over a wide frequency range.

Because the multivariate extension of EMD applied in this study is computationally very costly, we had to restrict our decomposition to 30 ICs. As mentioned in the methods section, extracting this number of ICs is still valid according available literature results. However, with higher-dimensional decompositions we would expect that cerebellar and subcortical ICs would have a much larger signal-to-noise ratio. But as our simulations with conservative, intermediate, and liberal sets of RSNs showed very robust and similar results, we are confident that using higher dimensional decompositions would merely strengthen our results.

Besides those limitations our method is not only able to extend the standard dFC to an frdFC approach, rather it also shows that merely looking at the separation of frequency scales by MEMD already reveals benefits in investigating brain networks. While brain graphs are traditionally based on thresholding correlation matrices, our approach offers a new way of simultaneously investigating the dynamics of such brain connectivity networks on different frequency scales. This is achieved by thresholding the static or dynamic correlation matrices calculated from time courses separated into different intrinsic modes. Thus, for each subject or session not only one static brain graph or one set of dynamic brain graphs can be constructed, rather brain connectomes can be resolved at various inherent frequency scales. This offers the possibility to adapt brain graphs to inherent and problem-specific time scales, and investigate graph theoretical properties like small-worldness, corresponding to different inherent dynamics simultaneously. This means that the emerging diagnostic values of functional connectivity in diseases like Alzheimer's disease and related dementias, schizophrenia or Parkinson's disease could benefit from a higher diagnostic sensitivity by looking at functional connectivity—and with it brain graphs—on different time scales. Also methods investigating cognitive states by relying on brain graphs Cribben et al. (2012) can adapt additional degrees of freedom by applying certain aspects of our approach.

In general we conducted our study on a very large dataset consisting of 400 sessions with 1115 windows each. Running clustering algorithms on such a huge dataset can only account for general features inherent to the data of all subjects—even if demeaning of the single correlation functions has been conducted. Such a dataset is suitable for a baseline study like ours, but it has to be replicated for smaller studies with a specific psychological hypothesis, if connectivity-states can also be extracted in a robust manner over scales, if the stability over scales also peaks at *k* = 4 or if scale-stability breaks down. If the latter turns out to be true, complexity-loss theory for systems under stress (represented by the stimulated brain) (Goldberger et al., 2002; Ahmed and Mandic, 2011) would be a plausible explanation.

Further studies could investigate modifications of our approach to apply MEMD on higher dimensional data. Since MEMD is computationally very costly with increasing number of channels, it would be desirable to develop a method for applying it onto finer parcellated cortices. Further studies could apply PCA on the dataset reducing dimensionality in the spatial domain. Then, on those reduced datasets, MEMD can be evaluated and the resulting PCs can be backreconstructed into the original dimension, but now frequency-resolved. Furthermore, future studies can delve into designing filter-banks and explore e.g., the feasible frequency resolution. Our provided repository of frdFC time courses is a suitable baseline for this purpose.

Another intriguing finding is that even in simulated time courses traversing artificial connectivity-states scale-stability is an inherent feature. Since the generating algorithms of those simulations are known, it could be possible to look for a deeper mathematical understanding of the scale-stability emerging from those operations. Thus, further studies could investigate the theoretical framework of scale-stability in the context of dFC and connectivity-states.

### 4.5. Conclusion

We introduced frdFC by means of MEMD and Butterworth filter bank investigations. Intriguingly, our frequency resolution of dFC revealed robust scale-stability of connectivity-states over a wide range of inherent frequency scales. This could be achieved by applying MEMD, which thus seems to be a suitable tool for a frequency-resolved level of dFC analysis. Furthermore, by decomposing time courses of ICs at an early stage of the analysis protocol, we were able to gain deeper insight into the behavior of connectivity-states, which otherwise could not be revealed. Thus our results suggest connectivity-state as a useful concept for cognitive studies and confirm that they are a stable construct, well adapted to a wide range of inherent and problem-specific time scales. Scale-stability was shown to offer a proper means to estimate the underlying model order, thus revealing the proper number of inherent connectivity-states extracted by clustering approaches and structurally stable across inherent dynamical processes on different time scales. *Post hoc* filter-bank studies show that scale-invariance of connectivity-states is a robust feature over more frequency-scales than offered by MEMD. But choosing an ill-shaped filter-bank can result in a mis-detection of the number of data-inherent states. Since MEMD is a data-driven approach, no fine-tuning is necessary for this purpose. Our novel approach to spectrally resolved dFC offers plenty of new degrees of freedom, in which pathological and healthy cognition may be distinguished.

To sum up more specifically (i) our simulated results point to scale-invariance of connectivity-states as being a valid quality criterion for model order selection and (ii) this finding encourages to look for a mathematically deeper understanding of the concept connectivity-state, since in simulated data the generating procedure is known. (iii) Finding similar scale-invariance structure in simulated and rs-fMRI connectivity-states for *k* = 4 is a strong hint toward this number of connectivity-states representing data inherent states. (iv) Our analyses show that scale-stability in both the simulated and rs-fMRI time courses is a robust and strong feature. (v) Choosing the natural way of detecting self-similar structures by using a data-driven approach like MEMD led to further frequency-resolution analyses via filter-banks, which confirmed self-similarity as a data-inherent feature detectable by frequency decompositions. Comprehending all findings yields the conclusion that connectivity-states as a concept are not just a multivariate, but also a multiscale entity, and that our novel approach represents a highly sensitive way of detecting data-inherent connectivity-states- or rather clustering structure.

## Author contributions

MG designed the approach and did the simulations and analyses. The paper was written by MG, AT, MWG, and EL. The study was supervised by MWG and EL. EL also contributed to study design.

### Conflict of interest statement

The authors declare that the research was conducted in the absence of any commercial or financial relationships that could be construed as a potential conflict of interest.

## Footnotes

^1^https://epub.uni-regensburg.de/35072/

^2^http://doi.org/10.5283/epub.32642
