# Supplementary material for: Frequency-Resolved Dynamic Functional Connectivity Reveals Scale-Stable Features of Connectivity-States
Source: Front Hum Neurosci. 2018 Jun 26;12:253. doi: 10.3389/fnhum.2018.00253 (PMC6036272; doi:10.3389/fnhum.2018.00253)
Supplement: Supplementary file 1 [file Image_1.PDF]

Supplementary Material: Frequency-resolved dynamic functional connectivity reveals scale-stable features of connectivity-states

Markus Goldhacker*, Ana Maria Tomé, Mark W. Greenlee, Elmar W. Lang
*Correspondence:
Author Name: Markus Goldhacker
markus.goldhacker@ur.de

1 SUPPLEMENTARY MATERIAL

1.1 Spectrum characteristics of ICs from gICA

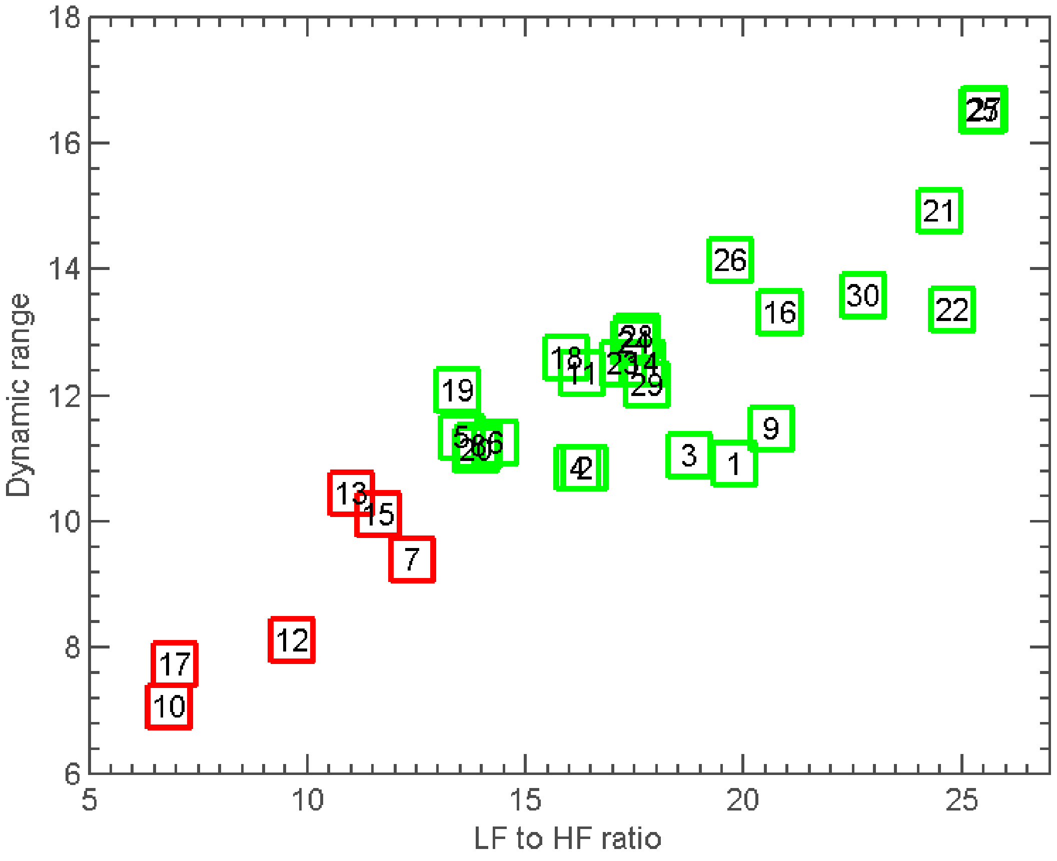

Figure S1. In this figure the low frequency to high frequency ratio and the dynamic range are plotted for each IC resulting from gICA (numbers from fig. 1A are depicted). ICs with red boxes are discarded in the conservative data set. ICs 7, 15, and 17 are artifact ICs and it can be seen that ICs 10, 12, and 13 have worse spectrum characteristics than the best artifact IC 7. Therefore using the conservative data set is most valid.

1.2 Correlation and covariance between IMFs of different indices

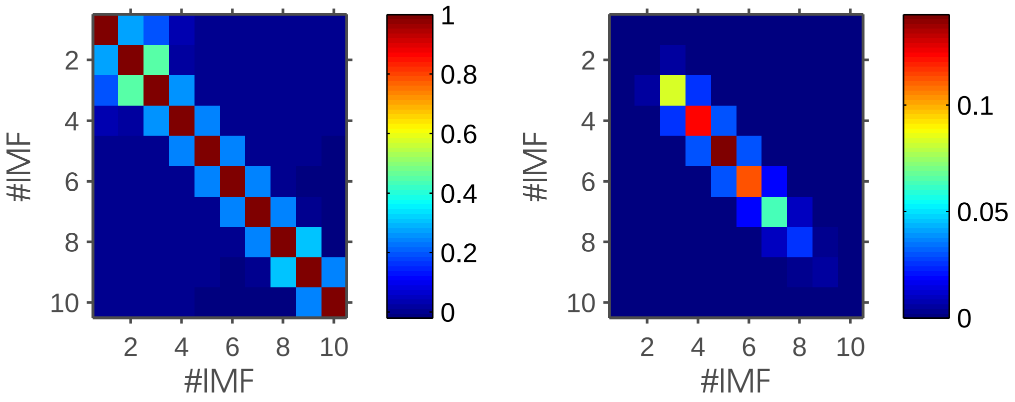

Figure S2. Depicted are the average correlation matrix (left) and covariance matrix (right) of IMFs over sessions and components. The low correlation and covariance values confirm the separation in very narrow frequency bands.

1.3 Scale-stability of connectivity-states for different k-means runs: original data

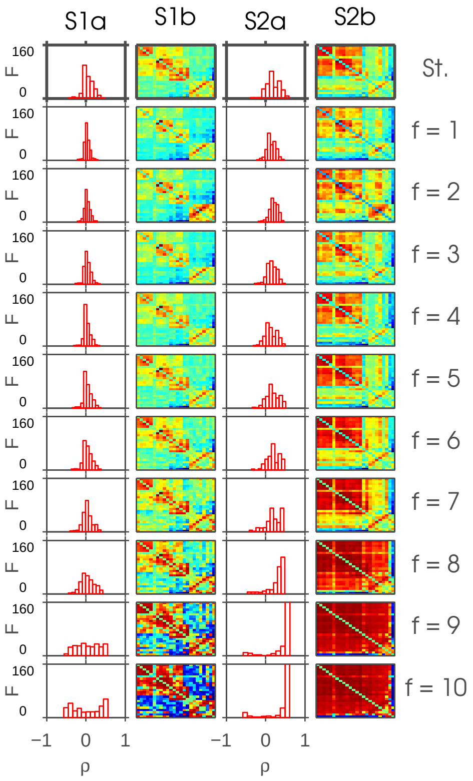

Figure S3. Depicted is the result of the ordering procedure on the k-means run with $k = 2$. Connectivity-states are shown in columns with suffix *b and the coloring is individually adjusted to range from minimum to maximum value to emphasize the structural similarity of connectivity states over frequency scales. The information of the distribution of correlation coefficients can be found in histograms plotted for corresponding connectivity-states in columns with suffix *a.

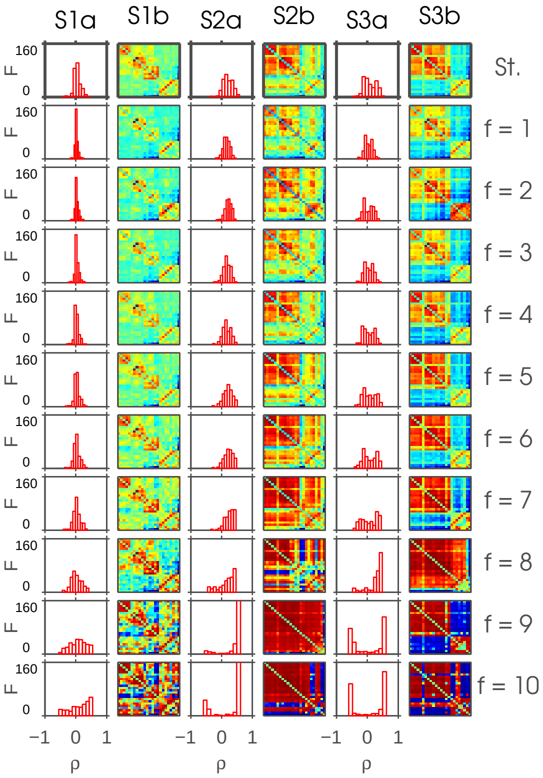

Figure S4. Depicted is the result of the ordering procedure on the k-means run with $k = 3$. Connectivity-states are shown in columns with suffix *b and the coloring is individually adjusted to range from minimum to maximum value to emphasize the structural similarity of connectivity states over frequency scales. The information of the distribution of correlation coefficients can be found in histograms plotted for corresponding connectivity-states in columns with suffix *a.

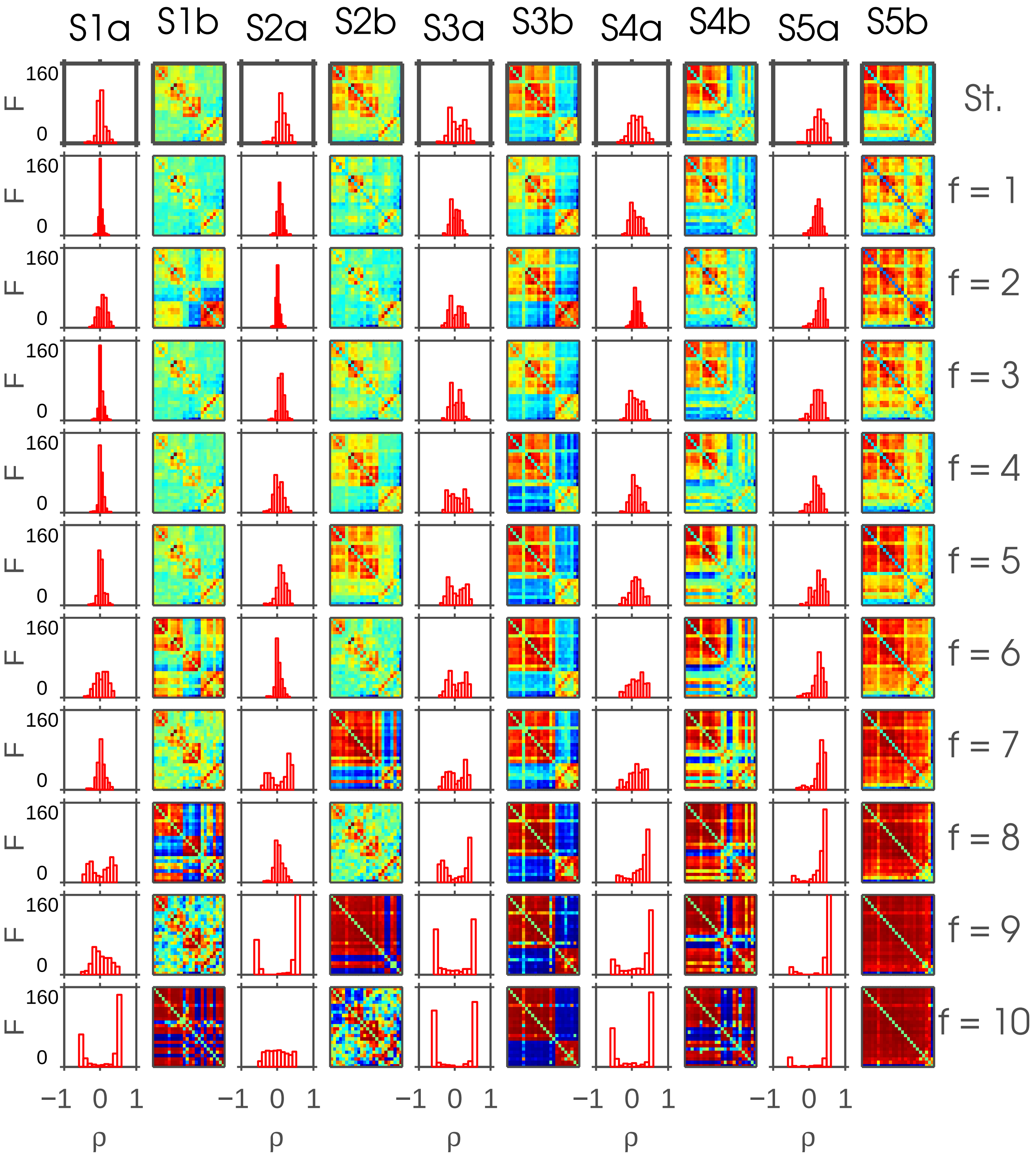

Figure S5. Depicted is the result of the ordering procedure on the k-means run with $k = 5$. Connectivity-states are shown in columns with suffix *b and the coloring is individually adjusted to range from minimum to maximum value to emphasize the structural similarity of connectivity states over frequency scales. The information of the distribution of correlation coefficients can be found in histograms plotted for corresponding connectivity-states in columns with suffix *a.

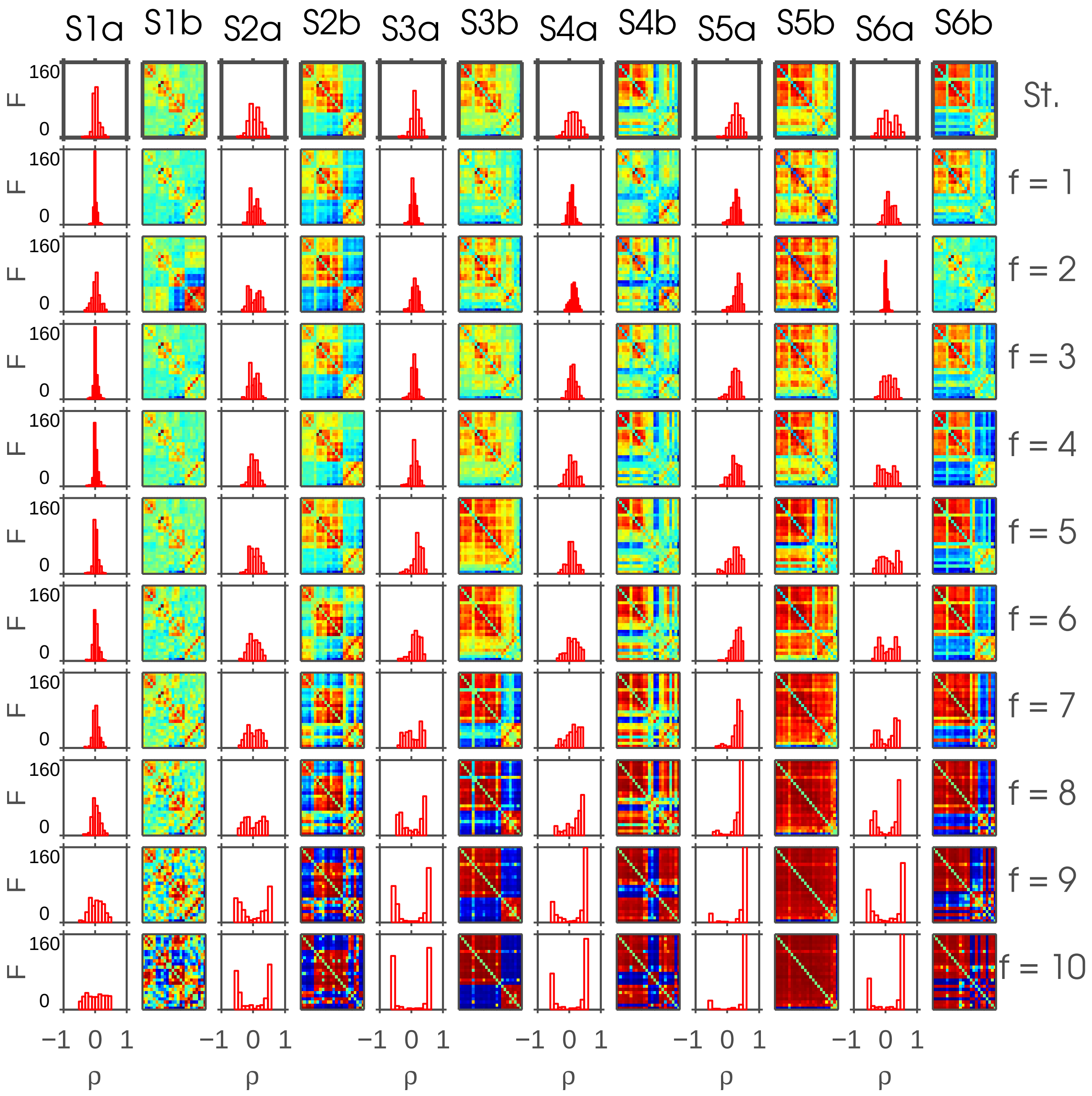

Figure S6. Depicted is the result of the ordering procedure on the k-means run with $k = 6$. Connectivity-states are shown in columns with suffix *b and the coloring is individually adjusted to range from minimum to maximum value to emphasize the structural similarity of connectivity states over frequency scales. The information of the distribution of correlation coefficients can be found in histograms plotted for corresponding connectivity-states in columns with suffix *a.

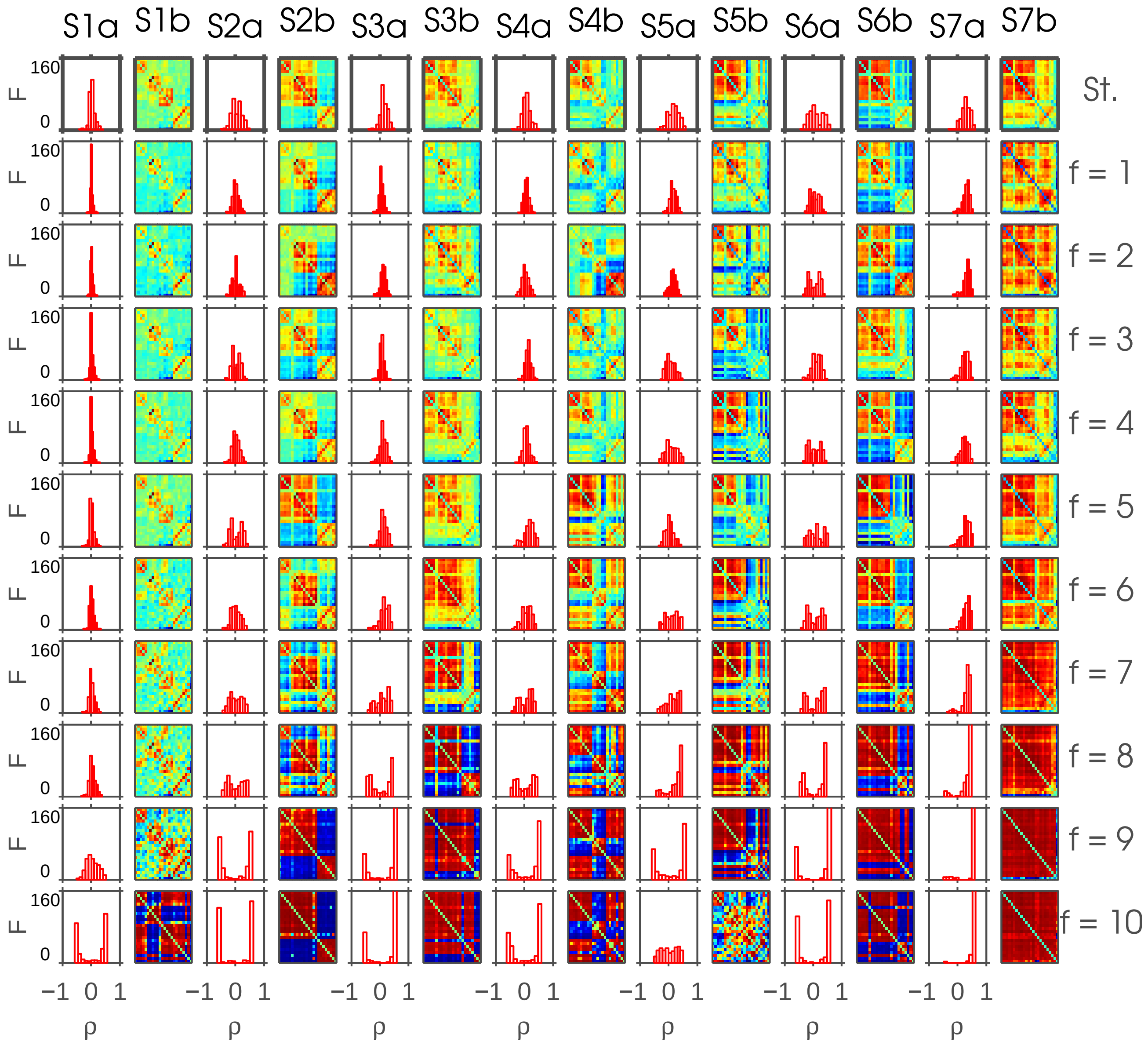

Figure S7. Depicted is the result of the ordering procedure on the k-means run with $k = 7$. Connectivity-states are shown in columns with suffix *b and the coloring is individually adjusted to range from minimum to maximum value to emphasize the structural similarity of connectivity states over frequency scales. The information of the distribution of correlation coefficients can be found in histograms plotted for corresponding connectivity-states in columns with suffix *a.

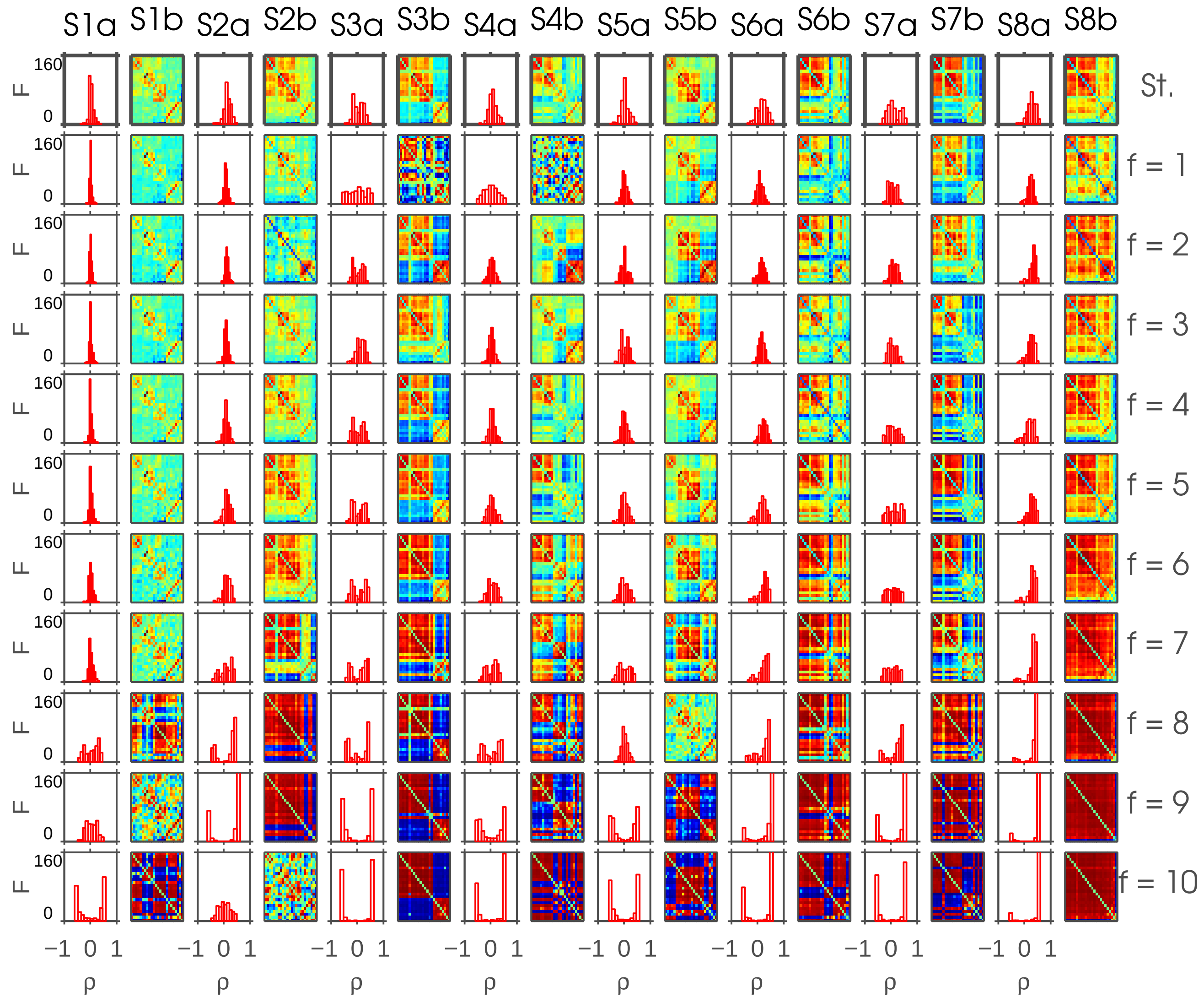

Figure S8. Depicted is the result of the ordering procedure on the k-means run with $k = 8$. Connectivity-states are shown in columns with suffix *b and the coloring is individually adjusted to range from minimum to maximum value to emphasize the structural similarity of connectivity states over frequency scales. The information of the distribution of correlation coefficients can be found in histograms plotted for corresponding connectivity-states in columns with suffix *a.

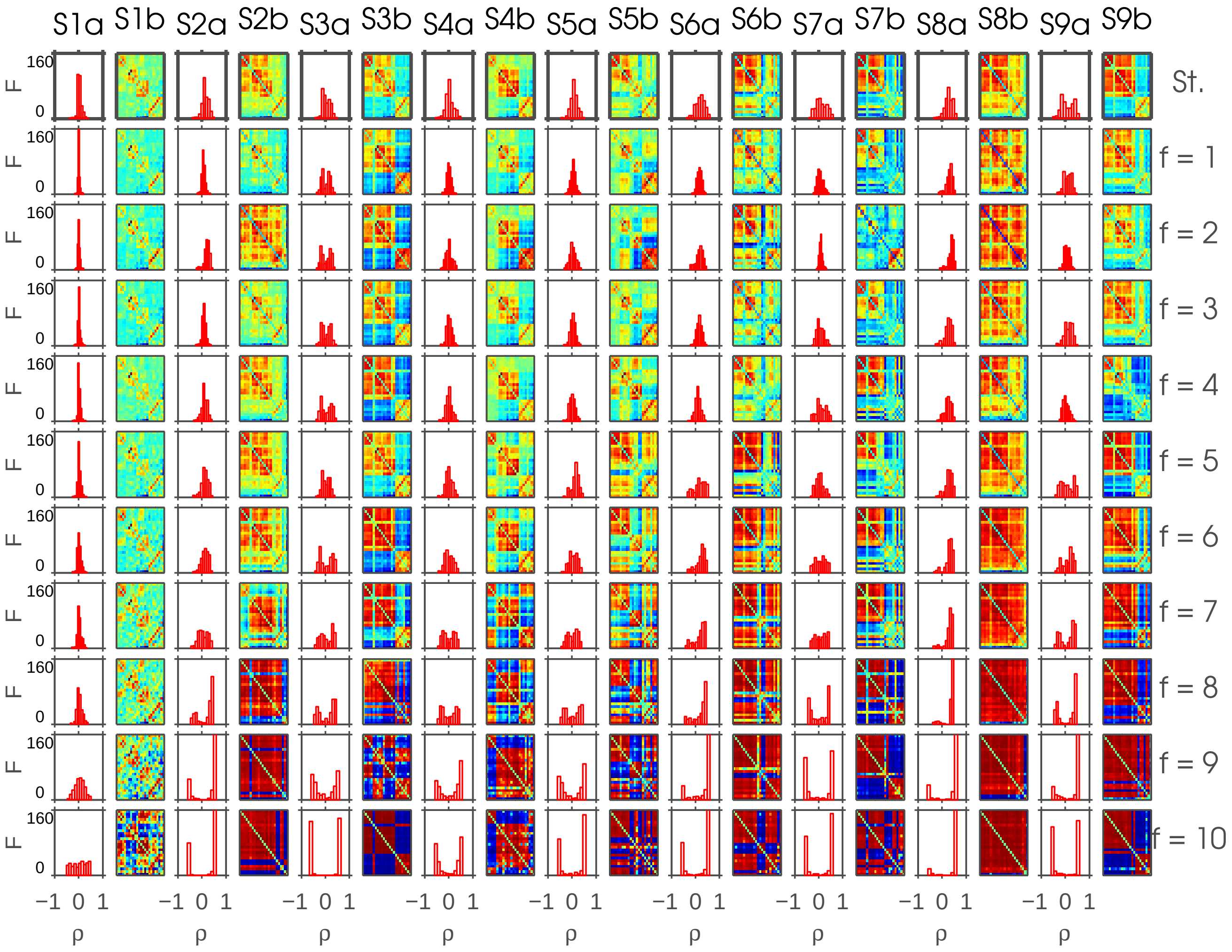

Figure S9. Depicted is the result of the ordering procedure on the k-means run with $k = 9$. Connectivity-states are shown in columns with suffix *b and the coloring is individually adjusted to range from minimum to maximum value to emphasize the structural similarity of connectivity states over frequency scales. The information of the distribution of correlation coefficients can be found in histograms plotted for corresponding connectivity-states in columns with suffix *a.

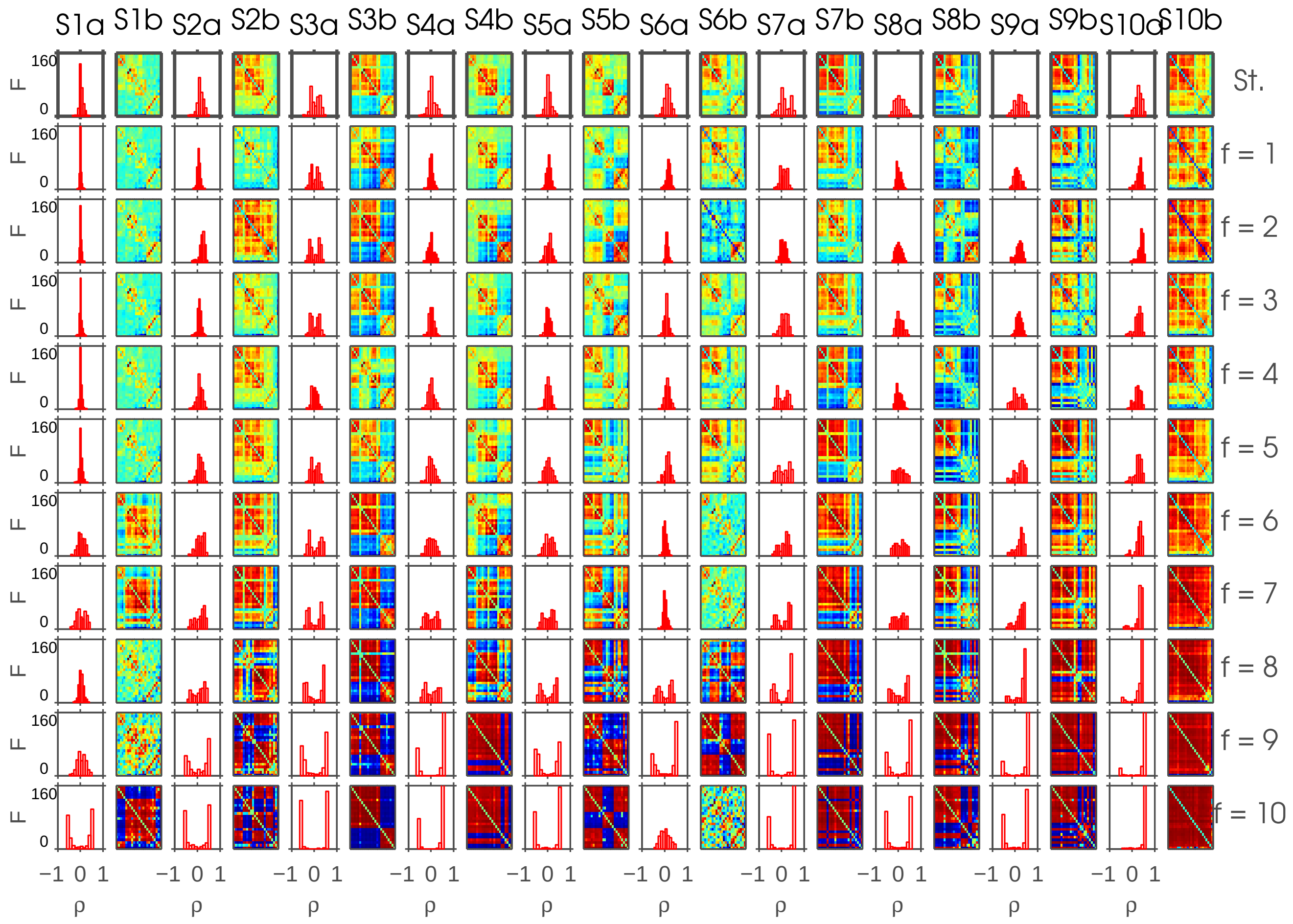

Figure S10. Depicted is the result of the ordering procedure on the k-means run with $k = 10$. Connectivity-states are shown in columns with suffix *b and the coloring is individually adjusted to range from minimum to maximum value to emphasize the structural similarity of connectivity states over frequency scales. The information of the distribution of correlation coefficients can be found in histograms plotted for corresponding connectivity-states in columns with suffix *a.

1.4 One exemplar of k-means applied on component-wise temporally demeaned dFC matrices

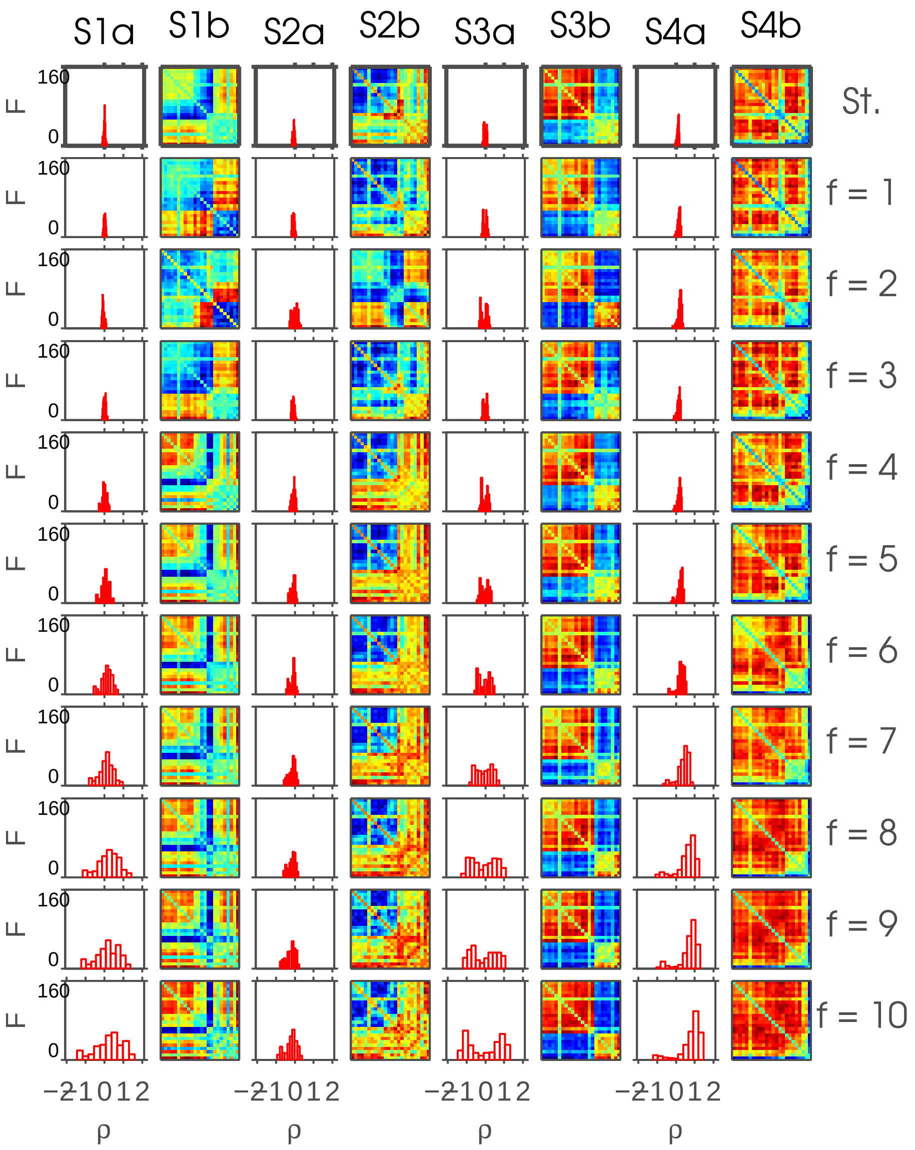

Figure S11. Depicted is the result of the ordering procedure on the k-means run with $k = 4$ applied on the demeaned version of the canonical approach. Connectivity-states are shown in columns with suffix *b and the coloring is individually adjusted to range from minimum to maximum value. The information of the distribution of correlation coefficients can be found in histograms plotted for corresponding connectivity-states in columns with suffix *a. Here eleven frequency scales are shown, since the MEMD algorithm extracted more IMFs than in the original time courses. This is due to the fact that shuffling introduces higher frequencies to the time courses.

1.5 Theoretically expected pattern of scale stability for $k_{inh} = 4$

Assuming there are k_{inh} connectivity-states inherent to the data and k-means identifies those k_{inh} connectivity-states as centroids in each run and also at each frequency scale f perfectly, then the behavior of $\langle\overline{I^{sim}_{\Delta f}}\rangle(k)$ can be deduced in this ideal case. Additionally, we assume that the cluster centroids that do not belong to connectivity-states express zero correlation with each other and with the connectivity-states. This considerations imply that the shuffling of frequency scales is redundant, because both non-connectivity-states and connectivity-states always align across frequency scales. Also averaging over different Δf is redundant, because each correlation either results in a correlation coefficient of $\rho = 0$ or $\rho = 1$. Therefore any Δf represents the sum equally and the sum over f in equation 2 can be discarded. With the perfect alignment of connectivity-states and non-connectivity-states across scales the different columns in the plot arrays can either be represented by $\rho = 1$ or $\rho = 0$. This means that in this hypothetical case $\langle\overline{I^{sim}_{\Delta f}}\rangle(k)$ reduces to

$$\langle\overline{I^{sim}_{\Delta f}}\rangle(k) = \begin{cases} 1, & \text{if } k \leq k_{inh} \\ \frac{k_{inh}}{k}, & \text{otherwise.} \end{cases} \tag{1}$$

Thus, theoretically, if k-means always finds the correct connectivity-states for each k and in each scale f, then $\langle\overline{I^{sim}_{\Delta f}}\rangle(k)$ stays constant until the desired number of connectivity-states is reached and then a drop-off with $\frac{k_{inh}}{k}$ occurs. The expected pattern from equation 1 is shown in figure S12 for $k_{inh} = 4$ inherent states. Intriguingly, a similarity to the evolution of the index in figure 4A1 can be seen.

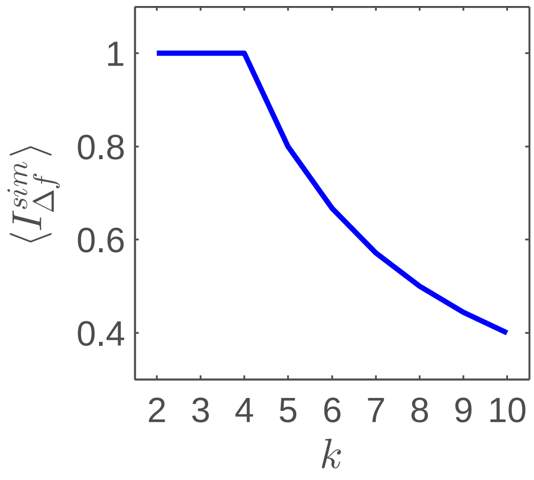

Figure S12. This figure depicts the theoretically expected behavior of the introduced scale stability index. The corresponding equation is deduced in section 1.5.

1.6 One exemplar of k-means applied on shuffled time courses over frequency scales

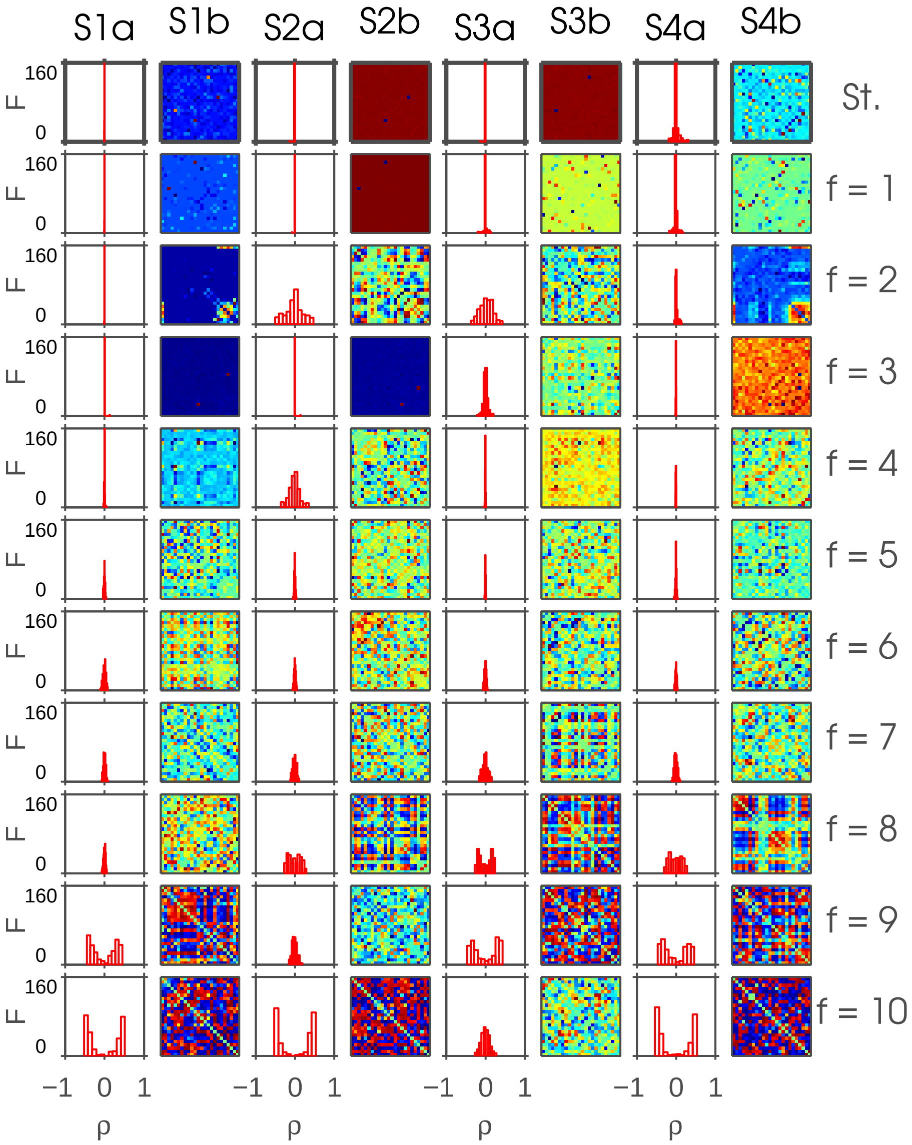

Figure S13. Depicted is the result of the ordering procedure on the k-means run with $k = 4$ applied on the shuffled version of the original time courses. Connectivity-states are shown in columns with suffix *b and the coloring is individually adjusted to range from minimum to maximum value. The information of the distribution of correlation coefficients can be found in histograms plotted for corresponding connectivity-states in columns with suffix *a. Here eleven frequency scales are shown, since the MEMD algorithm extracted more IMFs than in the original time courses. This is due to the fact that shuffling introduces higher frequencies to the time courses.

1.7 One exemplar of k-means applied on phase-randomized time courses over frequency scales

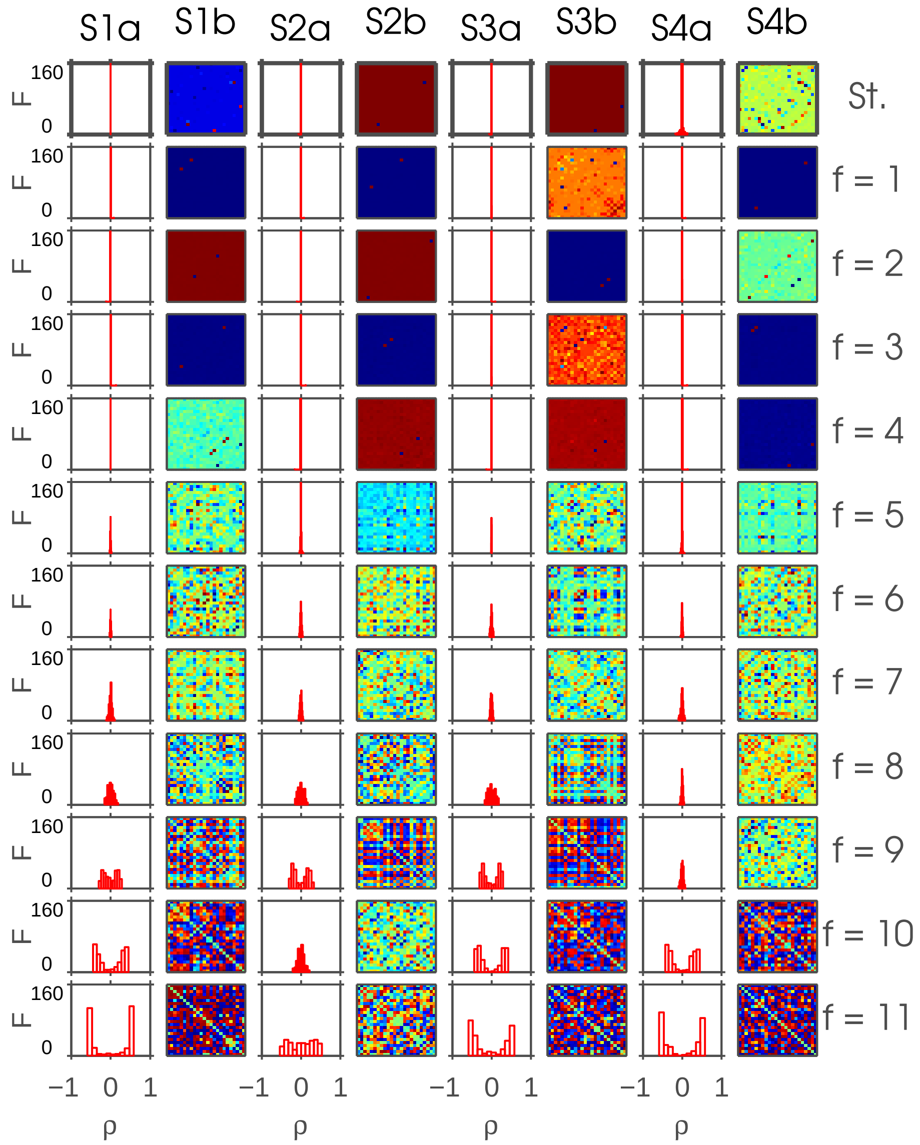

Figure S14. Depicted is the result of the ordering procedure on the k-means run with $k = 4$ applied on the phase-randomized version of the original time courses. Connectivity-states are shown in columns with suffix *b and the coloring is individually adjusted to range from minimum to maximum value. The information of the distribution of correlation coefficients can be found in histograms plotted for corresponding connectivity-states in columns with suffix *a.

1.8 Filter-Banks: simulated data

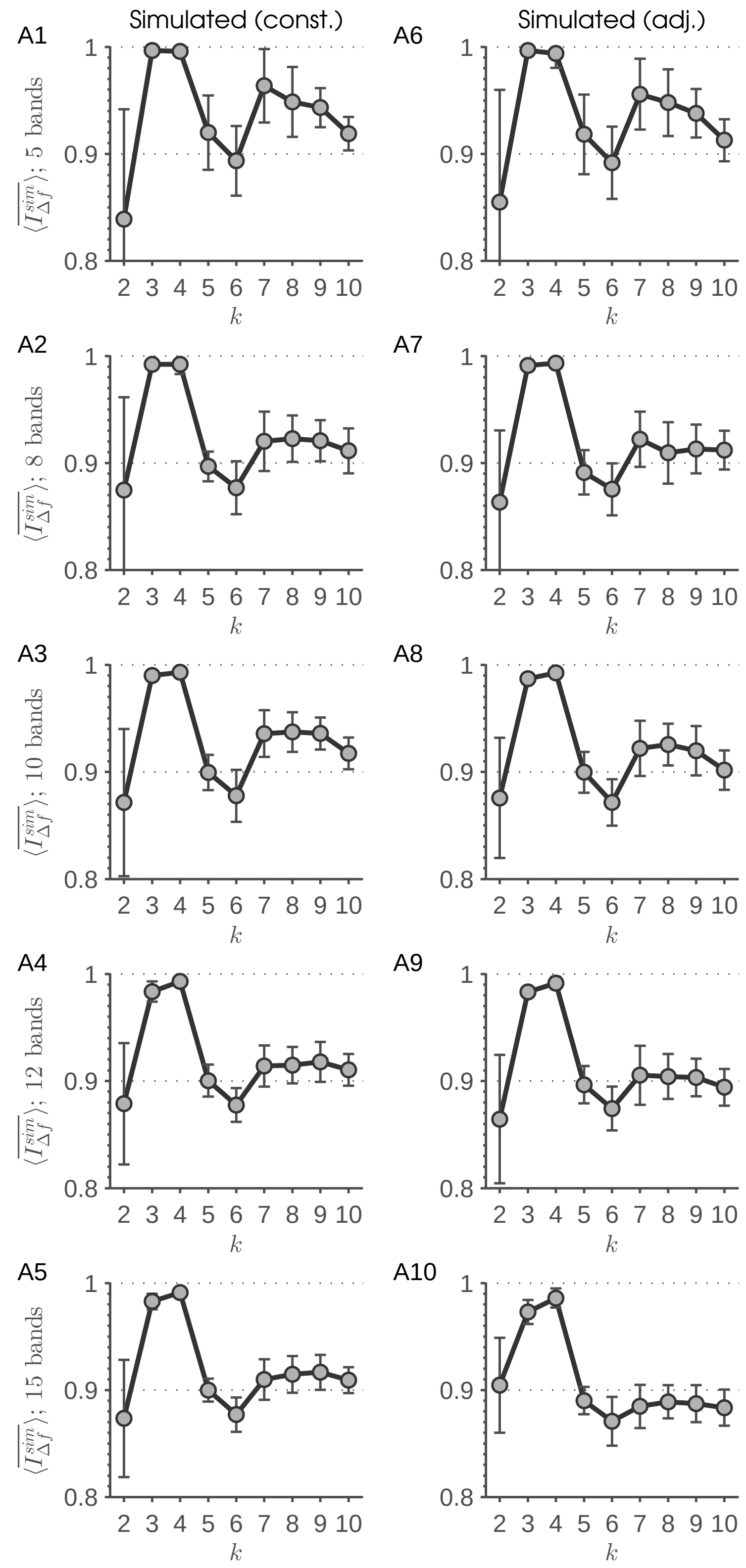

Figure S15. This figure summarizes the results of the scale-stability analysis for the filter-bank procedures with constant and adjusted filter order applied on simulated data with $k_{inh} = 4$.
